# Moving Forward: Understanding Correlates of Physical Activity and Sedentary Behaviour during COVID-19—An Integrative Review and Socioecological Approach

**DOI:** 10.3390/ijerph182010910

**Published:** 2021-10-17

**Authors:** Rachel L. Knight, Melitta A. McNarry, Liba Sheeran, Adam W. Runacres, Rhys Thatcher, James Shelley, Kelly A. Mackintosh

**Affiliations:** 1 Applied Sports, Technology, Exercise and Medicine (A-STEM) Research Centre, Swansea University, Swansea SA1 8EN, UK; 974302@swansea.ac.uk (R.L.K.); M.Mcnarry@Swansea.ac.uk (M.A.M.); 918800@swansea.ac.uk (A.W.R.); James.Shelley@swansea.ac.uk (J.S.); 2 School of Healthcare Sciences, College of Biomedical and Life Sciences, Cardiff University, Cardiff CF14 4EP, UK; sheeranl@cardiff.ac.uk; 3 Biomechanics and Bioengineering Research Centre Versus Arthritis, Cardiff University, Cardiff CF10 3AX, UK; 4 Institute of Biological Environmental and Rural Sciences, Aberystwyth University, Aberystwyth SY23 3FL, UK; ryt@aber.ac.uk

**Keywords:** physical inactivity, adults, coronavirus, older adults, sedentary time, movement behaviours, SARS-CoV-2, determinants, COM-B model, behaviour change

## Abstract

Population-level physical activity (PA) and sedentary time/behaviour estimates represent a significant public health issue exacerbated by restrictions enforced to control COVID-19. This integrative review interrogated available literature to explore the pandemic’s impact on correlates of such behaviours in adults (≥18 years). Five electronic databases were systematically searched in January 2021. Data extracted from 64 articles were assessed for risk-of-bias using the Mixed Methods Assessment Tool, with correlates identified, coded, and themed via thematic analysis. A socioecological model of during-pandemic PA was conceptualized and mapped to the Capability, Opportunity, Motivation, and Behaviour (COM-B) model of behaviour change mechanisms, which illustrates influences over five levels: Individual (biological)—general health; Individual (psychological)—mental health, cognition, motivation, and behaviour; Social—domestic situation, sociodemographic factors, support, and lifestyle choices; Environmental—resources and area of residence; and Policy—COVID-19-related rules. For sedentary time/behaviour, individual level factors, namely general and mental health, may be important correlates. Neither age or sex were clearly correlated with either behaviour. As we transition into a new normal, understanding which behaviour mechanisms could effectively challenge physical inactivity is essential. Targeting capability on a psychological level may facilitate PA and limit sedentary time/behaviour, whereas, on a physical level, maximizing PA opportunities could be crucial.

## 1. Introduction

Physical activity (PA) is well evidenced to benefit the general population [1], with small increases being positively associated with a decreased risk of premature all-cause mortality [1]. As one of the leading risk factors for non-communicable diseases, including cardiovascular disease, cancer, and type II diabetes, physical inactivity is predicted to be responsible for over five million preventable deaths per year [2]. Despite this, one in four adults globally do not meet PA recommendations [1]. Sedentary behaviour, defined as any waking behaviour characterized by an energy expenditure ≤1.5 metabolic equivalents (METs) while in a sitting or reclining posture [3], is an independent risk factor for mortality even among individuals meeting the PA guidelines [4]. Previous estimates suggest that adults spend approximately 60% of their waking time engaged in sedentary pursuits, equating to more than eight hours a day [5]. These estimates of population-level PA and sedentary behaviour represent a significant challenge for public health. Indeed, PA and reduced sedentary time/behaviour may be even more important with the emergence of the novel coronavirus disease 2019 (COVID-19) since being physically active is associated with a lower risk of community-acquired infections, including COVID-19 [6].

First described in December 2019, COVID-19, caused by being infected with the severe acute respiratory syndrome coronavirus 2 (SARS-CoV-2), was declared a pandemic by the World Health Organization (WHO) on 11 March 2020. As of 20 July 2021, there have been over 190 million confirmed cases and 4 million deaths associated with COVID-19 in 218 countries, areas, or territories worldwide [7]. In response to the emergence and transmission of COVID-19, the WHO issued advice for all countries to identify, manage, and care for new cases of COVID-19 [8]. Whilst the response was not heterogeneous globally, national responses included the introduction of social distancing, restrictions on travel, the cancellation of mass participation events, changes to work practices, and the introduction of self-isolation and quarantine to slow further spread, avoid overwhelming health systems, and to prevent infection among those at higher risk of severe outcomes [8]. Given the rarity of pandemics and the different approaches taken in response to the COVID-19 pandemic, our understanding of the effects of these restrictions on individuals’ lifestyles and health is limited.

Physical activity is a complex and multi-faceted behaviour; to fully understand the impact of the COVID-19 pandemic, it is necessary to explore the interactions between the individual, their social and physical environments, and relevant policies, which is consistent with a socioecological approach [9]. Socioecological models incorporate a broad range of variables that are expected to influence behaviour, and they can be used alongside other complementary, theoretically based models, such as the capability, opportunity, motivation, and behaviour (COM-B) model [10], to determine which conditions need to be met to facilitate behavioural change at an individual and ultimately population level [11]. The COM-B model outlines three potential mechanisms of behaviour change, each made up of two aspects: capability (physical and psychological), opportunity (physical and social environment), and motivation (reflective and automatic) [10]. The COM-B is the behavioural system positioned at the centre of the Behaviour Change Wheel (BCW), a framework that provides a structure to identify which aspects of behaviour provide suitable targets for interventions and which intervention functions are therefore most likely to be effective [11]. Assessing the impact of the COVID-19 pandemic on the correlates of PA and sedentary behaviour is essential to inform the response of policy makers and intervention designers seeking to increase PA and reduce sedentary behaviour to improve population health as we transition to and establish a new normal.

The aims of the integrative review were, therefore, to (i) interrogate the available literature to establish the impact of the COVID-19 pandemic on correlates of PA and sedentary behaviour conceptualized within a socioecological model and (ii) use the COM-B model to identify mechanisms of behaviour change directly mapped from the developed socioecological model to make recommendations to inform future PA intervention strategies and policy following the COVID-19 pandemic.

## 2. Materials and Methods

### 2.1. Literature Review Methodology

To inform the conceptualization of the socioecological model, an integrative review of both quantitative and qualitative literature relating to PA, sedentary time/behaviour, and COVID-19 was conducted in line with published guidance [12]. Both sedentary time and behaviour have been included to ensure no literature is excluded due to the absence of a measure of posture or specified behaviour. Electronic databases (EBSCOhost Medline, CINAHL plus, EBSCOhost SPORTDiscus, SCOPUS, Web of Science) were used to search key terms on 16 January 2021. Boolean and MeSH terms, developed following librarian guidance, were used to search for the following terms and variations of each term: “physical activity”, “exercise”, “sport”, “recreation”, “active travel”, “physical performance”, “physical function”, “sedentary time” “sedentary behaviour”, “sedentary lifestyle”, “physical inactivity”, “prolonged sitting”, and “coronavirus”, “COVID-19”, “SARSCov2”, “n-CoV”, and “novel coronavirus”. Original studies published in English that assessed correlates of PA and sedentary time/behaviour in adults aged 18 years or over during the COVID-19 pandemic were included. A full breakdown of article inclusion/exclusion criteria is provided in Table 1.

Two authors (RLK and AWR) independently reviewed all generated citations and abstracts to select eligible studies using Rayyan (QCRI, Qatar), coding articles as either “included” or “excluded”. Subsequently, all “included” articles at this stage were obtained as full-text articles and reviewed against the pre-defined inclusion/exclusion criteria independently by the two authors. Three disagreements regarding eligibility were resolved by discussion with a third reviewer (LS). For an example of the full search terms and a detailed outline of the study selection and data extraction procedures, see online Supplementary Material File S1.

### 2.2. Quality Assessment

Whilst a critical appraisal of the literature has not always been a core component of the integrative review process [13], it is now deemed crucial [12]. Therefore, the Mixed Methods Assessment Tool (MMAT) [14], suitable for assessing different study designs (mixed methods, qualitative, quantitative—descriptive, and randomized and non-randomized trials), was used to appraise the quality of included studies. Depending on research design, one author (RLK) independently rated five domain criteria as “Yes”, “No”, or “Unclear”, with a second author (AWR) randomly checking 25% of the ratings to ensure consistency. No discrepancies were identified. Each study was subsequently attributed an overall quality score, presented using asterisks (*) as a descriptor, ranging from 1*, where 20% of the quality criteria have been met, to 5*, where 100% of the quality criteria have been met [15]. No studies were excluded due to low quality.

### 2.3. Data Analysis and Model Development

Using the six-stage process of thematic analysis outlined by Braun and Clarke [16], one author (RLK) reviewed the data extracted from the retrieved literature to identify correlates of PA and sedentary time/behaviour during the COVID-19 pandemic. The initial coding process was deductively driven by the socioecological model of Sallis et al. [9], with codes allowed to emerge inductively from the semantic meaning of the data under the headings Individual, Social, Environmental, and Policy. Generated codes were categorized into sub-themes, named, and defined to accurately represent the data. During these stages, codes and themes were independently challenged by a “critical friend” (LS), checked back in reverse to the original data extracts, and, where necessary, refined to ensure congruity.

Utilizing the generated sub-themes, the first author (RLK) completed a two-step process: (i) conceptualization of the socioecological model consistent with Sallis et al. [9] and (ii) mapping of the developed context-specific model to the components of the COM-B [11]. Specifically, the COM-B model was deemed the most appropriate model to facilitate the exploration and understanding of the mechanisms of behaviour change conceptualized within the socioecological model. Moreover, the COM-B can be utilized in combination with the behaviour change wheel to identify which aspects of the behavioural systems need to be influenced and in what ways [10], thereby providing a framework to translate this information to inform future interventions and policy. To enhance transparency, credibility, quality control, and rigor [17], following the completion of each step, the “critical friend” additionally blindly cross-matched 10% of the studies against the generated model to ensure consistency in approach and that the data had been mapped appropriately. All discrepancies were discussed and reviewed in reverse, from the model to the original studies, until a consensus was reached.

## 3. Results

**Figure 1:**
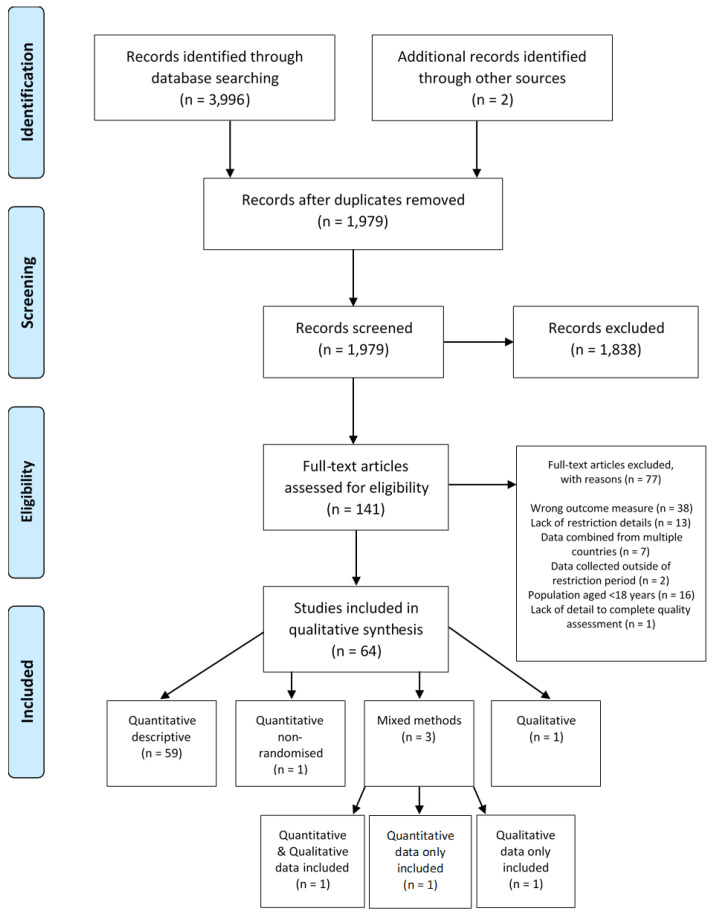
Schematic flow diagram of the integrative review process.

A total of 3996 articles were identified from electronic database searches, with a further two identified from secondary searches. Following the removal of duplicates, 1979 articles were screened, with 1838 excluded and 141 retrieved for full-text eligibility screening. Sixty-four articles were retained and included in the final analysis (Figure 1). The remaining articles encompass data from 155,313 adults aged 18 years or over [18,19,20,21,22,23,24,25,26,27,28,29,30,31,32,33,34,35,36,37,38,39,40,41,42,43,44,45,46,47,48,49,50,51,52,53,54,55,56,57,58,59,60,61,62,63,64,65,66,67,68,69,70,71,72,73,74,75,76,77,78,79,80,81] from 25 different countries, spanning six continents. All articles included >100 participants who were living under some degree of restrictions imposed to limit the spread of COVID-19 and presented data on correlates of PA, except for Kaur et al. [44] and Karuc et al. [42], which included 22 and 91 participants, respectively. Only 19 out of 64 (30%) provided details of correlates relating to components of sedentary time/behaviour. An illustrative summary of study details is provided in Table 2. Full, individual study characteristics and MMAT quality assessments are provided in Supplementary Tables S1–S3, respectively.

**Figure 2:**
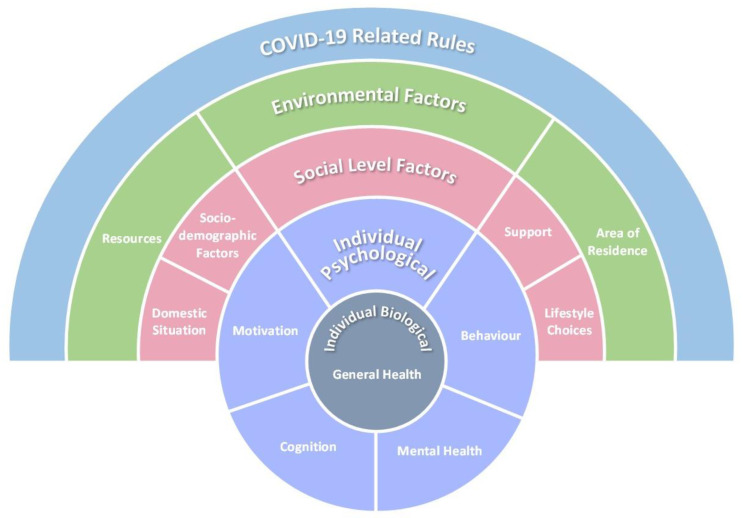
Socioeconomic model of correlates of physical activity during the COVID-19 restrictions.

A narrative synthesis of the findings, discussed in line with the dimensions of the socioecological framework of Sallis et al. [9], are outlined in the following section. Whilst these primarily relate to PA, where inferences to sedentary time/behaviour were possible, these are also noted. To help frame the impact, the findings from the analysis of the PA data were conceptualized into a socioecological model, Figure 2, that allows variables from different domains and the potential dynamic between individuals and wider influencing factors to be portrayed [9]. To aid with understanding and to align with evidence, only the strongest correlates were mapped. Due to the lack of robustness, consistency, and breath of data available relating to sedentary time/behaviour, the creation of a second, or combined, model was not deemed appropriate.

### 3.1. Individual—Biological Factors

#### 3.1.1. Age and Sex

For age, discrepancies in the definitions adopted to differentiate between and describe “younger”, “middle-aged”, and “older” adults limit the conclusions that can be drawn. Nonetheless, where younger adults aged <30 years were found to be more likely to increase their PA levels [25], those aged 18–25 years were more likely to be less active than those aged >25 years [65]. Furthermore, people aged 18–34 years or 35–54 years were more likely than “older adults” aged 55–74 years to be in a higher exercise category [33]. Whilst middle-aged adults (aged 40–64 years) were 1.2 times more likely to meet MVPA guidelines than their younger counterparts (aged 18–39 years) [43], those aged 43 years and over presented greater reductions in global guideline achievement [52]. Conversely, being aged 65 years or greater was also associated with maintaining sufficient [75] or higher [41] levels of PA. These findings are further complicated by reports that, in general, older individuals are more likely to exercise more frequently than younger (no age category specified) adults [30] and that age had no effect on either the change in PA levels [37] or behaviours [70].

There is little consensus in the literature as to the influence of sex on PA levels during the first wave of the COVID-19 pandemic. Specifically, where sex differences in PA levels were observed, females were reported to be more likely to be more active [38,75], to increase their PA levels [28,47,72], or to have smaller reductions in PA levels [28,52]. In contrast, others reported sex differences that favoured males [43,51,61,66,70], whilst some found no sex differences [18,30,37,42,57]. Additionally, in one instance, the difference between males and females was only apparent for light PA and not moderate, vigorous, or moderate-to-vigorous PA (MVPA) [26].

Similar variations in findings were observed for sedentary time/behaviour. Age may play a part in this complex depiction; indeed, screen-time habits declined with increasing age [32]. Nevertheless, being a younger adult was associated with being more likely to increase overall sedentary [28] and sitting time [65] but also a decrease in screen time obtained from watching television [19]. Regarding sex differences, where overall sedentary time [28] and, conversely, having more active breaks [66] were both reported to be higher in men, sitting time increased irrespective of sex [66,72]. However, further findings indicated that sitting time was higher in females [26,65]. For screen time, there were contradictory findings reported, with both males [19] and females [32] being the most likely to increase time spent watching television. There is some suggestion, though, that such differences may be attributed to the type of screen time engaged with or reported. Where television time (and internet use) was higher in females, more males reported an increase in video-game use [32].

#### 3.1.2. General Health

Multiple variables associated with general health present as factors that positively or negatively influenced PA and sedentary time/behaviour. Lower perceived overall general or physical health has been related with being significantly less active [63,65,69]. More specifically, negative associations were identified between PA and body mass [66,69], physical and general fatigue [24], sleep quality [40,80], and having a chronic or high-risk health condition [36,43,69,70], whilst not meeting guidelines for light-intensity PA [26] and spending less time per week being physically active [72] were linked with body mass index (BMI). Positive associations were found with higher perceived general and/or physical health and PA [18,24,31] and outdoor versus indoor exercise [32]. It is, however, pertinent to note research that highlighted no association between BMI and change in PA levels [37] and significantly higher levels of physical inactivity among individuals without compared to those with a chronic disease [65]. Similar findings were also observed in sitting time [65,72]. Negative associations were also found between sleep quality, television/computer/tablet use [80], and sitting time [54], perceived health and sitting time [79], BMI and screen time [19], and physical fatigue and sitting time [24]. Positive associations were observed between physical health and sitting time [18,65] and general health and screen time [32], with an inverse association reported between body mass and taking active breaks [66].

### 3.2. Individual—Psychological Factors

#### 3.2.1. Mental Health

Multiple associations were identified between components of mental health and well-being and PA. Although some of the evidence within this theme is of lower quality [31,56,57,59,60,71,73,74], it remains clear that having a better overall mental health status is associated with being more physically active. This is demonstrated with relation to walking [18], total volume of PA [18], light-intensity PA [24], moderate-intensity PA [21], vigorous-intensity PA [21], MVPA [26,41], general PA levels [49,57,59,61,63,69,73,78], and outdoor PA [49]. It is also pertinent to note that the correlation between overall mental well-being and PA may be stronger in females than males [57].

Correlations were identified with anxiety, depression/mood, and emotions. For anxiety, higher levels were associated with decreased or less PA [23,35,41,56,61,71,74,80] and outdoor activity [49], whilst lower levels were associated with participating in physical exercise [48,74] and achieving recommended PA guidelines [53]. Further, non-directional, significant interactions were also reported [26,63]. However, not all results supported these findings, with non-significant differences observed for generalized anxiety between active and inactive individuals [49] and severe anxiety having a stronger association with higher MVPA than moderate anxiety [61]. For depression/mood, positive associations were observed between lower depression/mood levels and engaging in physical exercise [46,74], the volume of MVPA [51], maintaining or slightly increasing pre-COVID-19 PA levels [40], meeting PA guidelines [53], and moderate- (over vigorous-) intensity PA [29]. Higher levels of depression were linked to changes in pregnancy exercise routines [39], whilst having high levels of both depression and anxiety almost doubled the likelihood of being less physically active [71]. Further non-directional associations were also reported [26,34,63,68]. For emotions, relationships between higher stress levels and decreased [35,47,59], less [59,60], or non-participation in [74] PA were reported. Additionally, poorer overall emotional well-being [61] and feelings of sadness [80], loneliness [59,80], and distress [57] were all reported to be detrimental to levels of PA.

With regard to sedentary time/behaviour, correlations were identified with components of mental health. Sedentary time [21] and screen time [21,59] were negatively associated with overall mental health. Higher levels of depression were associated with increased screen time [59] and sitting time [59,72]; higher levels of anxiety with increased screen time [80] and sitting [72]; and emotions, incorporating loneliness [59,80], sadness [80], and higher levels of stress [59] with increased screen time. However, additional findings showed no association between any emotional states and sitting time [59], depression and sedentary time [51], or parameters of mental health (self-perceived, depression) and sitting time [59]. Nonetheless, interactions were observed between mental health, PA, and sedentary time/behaviour, with better mental health status and higher levels of PA associated with daily sitting time [18] and lower increases in screen time [32].

#### 3.2.2. Personality Traits

Minimal evidence, mostly of low quality [77], infers that overall [77] or components of personality may have influenced PA levels and sitting time. Higher levels of neuroticism were associated with being less active [67,77] and sitting more [77], whilst being more extroverted (including activity-extraversion), conscientious [67,77], and/or agreeable [77] were related to higher mean levels of PA and decreased sitting time. Being more open was related to being more active but was unrelated to sitting time [77].

#### 3.2.3. Motivation

Stemming from multiple different conceptual elements, during the COVID-19 pandemic, motivation also presented as a strong correlate of PA. On an intrinsic level, autonomous motivation was related to being more active [49,61,76]. Emotional and psychological well-being [20], perceived benefit [49,61], maintaining good health [20], feeling better about oneself [20], affective judgements [67]—particularly enjoyment [20,49,61], the level of interest [33], desire to participate, and importance placed on PA [20] were all identified as potential influential PA motives. Additionally, where positive affect was positively related to MVPA [26,55] and, in some instances, moderate-intensity PA [26], negative affect was negatively related to MVPA [55]. On an extrinsic level, external regulation [49], striving to achieve goals [67], and introjected factors, for example, forcing oneself or viewing PA as a drudgery task, were associated with PA regulation [20,61]. Conversely, being amotivated [49], or having a general lack of motivation, was related to being less active [44,47].

#### 3.2.4. Cognition

Physical activity modulation has been linked to cognitive characteristics. In adults, correlations were observed between confidence [49,61], identity [67], perceived capability [76], resilience factors (locus of control/self-efficacy/optimism) [26,27], knowledge [43,56], and PA levels. However, no association between knowledge and behaviour was reported [76]. It is unclear whether specific COVID-19 concerns impacted engagement; whilst a fear of contamination was a reported concern [33], it served as both a PA driver and inhibitor [25].

#### 3.2.5. Behaviour

Actions and responses, or behaviour factors, had important repercussions for PA during the COVID-19 pandemic. Higher levels of pre-restriction PA were linked to having a higher probability of maintaining, increasing, or having sufficient levels during the restrictions [30,75] but also related to having the greatest declines [28,37,42,57,78]. Larger reductions in PA were observed in adults who previously attended the gym [37], exercised with friends [33], or engaged with a sports club [33,42]. Merely participating/being previously active and therefore having an established habit had positive effects on PA levels [23,30,31,42,67], including time spent engaging in outdoor activity [49], and led to being more likely to achieve PA guidelines [38]. However, trends were observed whereby adults classified as “less active” before COVID-19 actually also increased the time they spent being physically active during the period of restrictions [28,57].

Whilst an association with behaviour is apparent, the mechanisms of effect are potentially complex. Relationships were reported between behavioural intention and PA levels [72,81]. However, additionally, associations were reported between prior PA habits, intention, and autonomous motivation during the pandemic [31], with such social cognition constructs (autonomous motivation, perceived behaviour control, attitudes, subjective norms) potentially mediating the relationship between past behaviour and subsequent intention [45]. Similarly, associations were observed between behavioural planning and PA levels [61,67], with planning also identified as a potential mediator between past behaviour and intentions [45].

### 3.3. Social Level Factors

#### 3.3.1. Sociodemographics

A general association with income was observed [41,67], with higher income related to a higher exercise frequency [30], achieving sufficient [75] or increased levels of PA [37], and being more likely to change to more intense PA [70]. Conversely, having a lower income was related to lower PA levels [69], with COVID-19-related changes to income associated with a higher risk of greater declines in PA [37] and changes to pregnancy exercise routines [39]. Being food secure, potentially related to income, was also negatively related with sitting behaviour [79]. However, being from a higher socioeconomic status family was found to be a predictor of both physical inactivity and sitting time [65].

Adults with a higher level of education were less likely to decrease their PA levels [25,33,52], with education being positively correlated to MVPA [67]. However, these findings are counterbalanced by reports of no significant association [30,43] and physical inactivity being significantly higher among those educated to graduate level or above [65]. Furthermore, being a student was, in general, related to being less active in comparison to pre-COVID-19 [40], significant decreases in MVPA [42], decreases across all PA intensity levels [28], and higher levels of physical inactivity [65] and sitting time [65,72]. No association was found between student living environment (university residence, shared apartment, with family) and sitting time [72].

Regarding employment status, where in some instances a general association was observed [67], and employed individuals showed significantly lower reductions in PA levels [52], being unemployed was equivocally related to decreased [37], insufficient [43], or higher levels of PA [41]. Where those who transitioned to working at home during the pandemic increased their PA, those who did not or were already working from home experienced a decline [37].

Whilst ethnicity may be related to variations in parameters of PA [34,69,79] and sitting time [79], the breadth of data on which to draw inferences is limited.

#### 3.3.2. Support

Having better social relationships was related to higher levels of moderate- and vigorous-intensity PA [50], whilst lower perceived social well-being was associated with engagement in less PA [61]. Access to less social support was related to being less active [49,59,61], with this suggested to be particularly pertinent in adults who were already classed as inactive [49]. However, no link was found between social opportunity and different PA modalities (i.e., for transport, at work, in the neighbourhood) [76]. A lack of access to structured support from instructors [44], organized activities, friends/companions, and the competitive aspects of exercise [33] were all deemed detrimental. Indeed, a degree of association was also observed between being able to engage in PA with others and mental health [49], an already noted, potentially important correlate.

#### 3.3.3. Domestic Situation

Parameters of an adult’s home life, or domestic situation, were thematically highlighted as potential PA facilitators and barriers. Living alone was associated with greater decreases [37] or starting to do less intense PA [70], whereas although some reported no effect of having dependents at home [37], others reported that having children was associated with greater increases in PA [47,67] or starting to do more intense PA [70]. Furthermore, although the volume of PA increased as the number of children per household increased [79], the reverse was observed for the number of grandchildren [23]. Having a partner or family to exercise with [33] (particularly for females [22]), a dog [62,67], being married [41], a housewife [65], or living with a nuclear but not joint family [65] were all related to higher PA levels as opposed to being single, which was associated to higher levels of physical inactivity [65]. However, living with a nuclear family predicted higher levels of sedentary behaviour (sitting time) [65]. For women, having stable childcare provision positively impacted opportunities for PA, whilst increasing childcare demands were linked to decreases in confidence and more difficulty engaging in PA [61].

#### 3.3.4. Lifestyle Choices

Associations were identified between choices regarding other health-related behaviours and PA, specifically diet. Reducing food intake was associated with increases in PA [25], a negative correlation was observed with pre-prepared food or snack intake [60], a positive correlation with general changes to diet [40], and significant differences (direction unspecified) with not eating a Mediterranean diet [72]. For sedentary behaviour-related outcomes, alcohol consumption, eating a Mediterranean diet, and/or being a non-smoker were related to increased sitting time [72], whilst taking active breaks afforded some protection over poor dietary choices [66]. Significant correlations were noted between PA and sedentary time/behaviour. Being less sedentary was related to being more active [33] and vice versa [54,65,79], with adults who were more active pre-restrictions potentially being more likely to report the highest increases in sedentary time [28]. However, no specific correlation was identified between stage of change (PA) and sitting time, with increases observed with groups in the contemplation, preparation, action, and maintenance stages [72].

### 3.4. Environmental Factors

#### 3.4.1. Area of Residence

Whilst acknowledged as one of the weaker themes identified, a potential association was identified between factors relating to an adult’s area of residence and PA levels. Living in an urban or metro area was related to undertaking less PA [65], being less likely to meet MVPA guidelines during the pandemic [43], being more likely to report pregnancy exercise routine changes [39] and increased sitting time [65]. Additionally, not having access to outdoor space was linked to starting to do less intense PA [70]. However, with other reports of no significant effects of any neighbourhood environment variables on PA [67], the magnitude of importance of area of residence remains unclear.

#### 3.4.2. Resources

With the enforcement of restrictions came a loss or change in access to resources, including facilities and equipment. Access to sports clubs [33], gyms [44,54], and suitable (gym) equipment [51] represented a major obstacle to engaging in PA. Having access to equipment at home was related to being more active [67] and predicted greater levels of PA, planning, and autonomous motivation [45]. Purchasing home equipment also attenuated declines or led to increases in PA [37]. The effects of having access to cardiovascular and/or strength training equipment were potentially mediated by and correlated with autonomous motivation [45], with autonomous motivation and components of the theory of planned behaviour (attitudes, subjective norms, perceived behavioural control) also potentially mediating the relationship between equipment availability and PA intention and/or habit [45]. Engagement with alternative resources, specifically technology-driven, virtually delivered fitness platforms, (i.e., exergaming, online classes), led to increases in [37] or higher levels of total PA [33] (compared to those who did not) or the maintenance of PA routines [44,56]. Additionally, the use of a specific PA app and its gamification features was related to more positive changes in PA but not sedentary behaviour (sitting time) [81].

### 3.5. COVID-19-Related Rules

Whilst the country-specific COVID-19-related rules and regulations that were implemented to curtail the spread of the virus may have had overarching, more indirect, negative effects on adults’ PA (as identified in the previous themes), the direct effects were variable. Although in some instances, social distancing measures had a negative effect on MVPA [67], in others, no specific effects of lockdown policy or COVID-19 restrictions were observed [43,49], or the restrictions presented barriers to PA for females but not males [61]. Similarly, being furloughed was associated with greater declines in PA [37], transitioning to working from home with increased PA [37], and changes to work status (working from home or lost job) had no effect [58,61]. Conversely, such changes were related to higher sitting time (working from home or lost job) and screen time (lost job) [58].

Other changes to routines also had varying effects. Whilst some found that more time was available, which facilitated PA opportunities [33,47,54], others found reductions in time to be a barrier [33,54]. Not being able to continue and missing usual exercise regimes was related to less PA [33,61], whereas those who were able to adapt their routines were able to limit their PA declines [37,56]. The specific limitations through a perceived lack of opportunity to be active also had negative connotations for PA [49,61,76].

## 4. Discussion

This review sought to explore the correlates of PA and sedentary time/behaviour in adults aged 18 years or over during the unique period of enforced lifestyle restrictions during the COVID-19 pandemic. A recent systematic review found that in the vast majority of included studies PA decreased, and sedentary behaviour increased in both adults and children [82]. Enhancing our understanding of the multilevel influences on PA and, where possible, sedentary time/behaviour is therefore urgently needed to effectively guide future public health initiatives and policies.

For PA, the model illustrates potential influences over all five levels: Individual (biological), Individual (psychological), Social, Environmental, and Policy. For sedentary time/behaviour, the findings provide some indication that individual level factors, namely general and mental health, may be the primary correlates of importance. Indeed, it is already established that the relationship between mental health as the overall concept or as specifically defined conditions (i.e., depression, anxiety) and PA/sedentary behaviour is bi-directional [83]. Specifically, poor mental health status often leads to being less physically active and more sedentary, whilst being less active and engaging in more sedentary behaviours can have negative implications for mental health [83]. Several studies have reported this to be a significant issue during the first stage of lockdown restrictions [84]. Nonetheless, more detailed discussions of this correlate are precluded by the lack appropriate available evidence and indeed robustness during the COVID-19 restrictions.

Prior behaviour and, more specifically, habits were associated with PA engagement during the periods of restrictions [23,28,30,31,37,42,57,67,75,78]. It is, however, apparent that relationships and interactions between factors from different levels of the socioecological model and the magnitude of effect that these may have at an individual level may, at least in part, explain some of the variations in the behaviour observed. The ability to maintain habits was, for some, directly influenced by a loss of access to resources and facilities [33,37,42]. For individuals who participated in team sports [33,42] or utilized gyms [37] or other sporting facilities (e.g., swimming pools) to keep active, pre-COVID-19 participation and habit could have become irrelevant given that the opportunity had been removed. In contrast, such impact on habits were less manifest for those who engaged in outdoor physical activities, such as running. Whilst it could be argued that being physically active and less sedentary does not have to be dependent on equipment, establishing new habits may be challenging if sociodemographic situations [37,39,43,69], support structures [33,44,49,59,61], and/or local infrastructure [65,70] are not optimal. Notwithstanding these factors, individuals may also need to draw on and maintain their personal motivation on an intrinsic [20,26,33,49,55,61,67,76] and/or extrinsic level [20,49,61,67] and believe in their own capability [26,27,43,49,56,61,67,76].

Interestingly, unlike pre-COVID-19 [85,86], during the pandemic restrictions, neither age or sex presented a clear correlation with either PA or sedentary time/behaviour. It is, however, pertinent to acknowledge other factors that may have influenced these findings. As outlined in Table 2, the countries in which the studies were undertaken and the level of restrictions imposed, even sometimes within countries, varied significantly. Additionally, seasonal differences, which are already known to impact both PA and sedentary time/behaviour [87], were not accounted for. Individuals surveyed who resided in countries where the weather facilitated outdoor activity may not have been as severely impacted by any imposed restrictions. Finally, studies predominately reported levels of MVPA. Where light-intensity PA was reported, sex differences were found, with females being more likely to engage in sufficient levels in comparison to males [26]. Given that even small increases in PA can have positive benefits [1], with a move towards 24-h movement guidelines [88], this finding warrants further exploration.

It is apparent that there may be differences in the level of impact different correlates have for different age or sex groups, such as mental health having a greater impact on PA in women [57], with age-related differences in the type of screen time that needs to be challenged [32]. However, there were insufficient group-specific data, which precluded further interpretation. Such differences, however, are theoretically not unexpected. If, pre-COVID-19, different populations (i.e., older adults) had different motivators and barriers to PA [89,90] that require different batteries of behaviour change techniques to facilitate change [91], then it stands to reason that the correlates of their behaviour during these periods of “unknown” could be different. Only three studies specifically surveyed adults aged ≥60 years [26,68,78].

### 4.1. Recommendations for Policy: Mapping to the COM-B

Understanding which mechanisms of behaviour need to be targeted to develop effective interventions or strategies to facilitate PA is essential. Mapping the strongest identified correlate themes for PA to the components of the COM-B [11] (Table 3) highlights that, to some degree, changes to all behavioural components could be needed. However, when considered in context with the strength of evidence supporting each theme, as previously discussed, and the frequency of component identification, capability (psychological) and opportunity (physical) become the core focus for attention. Whilst it is clear that the removal of physical opportunity had a significant impact on PA levels during the initial pandemic control restrictions, future policies need to not only consider this but that the application of strategies that promote psychological well-being may be vital, both of which are not mutually exclusive.

During the easing of restrictions, particularly within the United Kingdom, sports and leisure facilities were amongst the last to re-open. Moreover, as of June 2021, some facilities had not yet re-opened at all, with others having a significantly reduced capacity. The benefits of PA for health and well-being have been deemed irrefutable [92]. Therefore, if measures are not taken to facilitate at least a return to access at pre-COVID-19 levels or improve access to alternative options (i.e., outdoor gyms, cycle tracks), then, especially in more rural areas where opportunity is already limited, the negative repercussions, not just immediately but for future generations, could be extensive. Moreover, given the observed correlations with sociodemographic-related factors, limiting access to affordable PA options will only serve to widen the current socioeconomic health gap.

### 4.2. Strengths and Limitations

Despite the rigorous, systematic approach adopted, underpinned by published guidance and the use of validated tools, this review is not without limitations. In all epidemiological research, the results will always be partially dependent on who chooses to participate and the variables that the studies chose to explore. The data included in this analysis are cross-sectional. Therefore, even where a direction of effect has been stated, this only infers correlation, not causation. The majority of data were collected via self-report measures, with retrospective recall of pre-COVID behaviour patterns. During the unprecedented COVID-19 situation, it does, however, have to be accepted that these online methods, even with their potential accuracy and generalizability limitations [93], ultimately provided the most appropriate approach. It is also important to note that (i) 17 studies used unvalidated measures of PA or sedentary time/behaviour [19,25,30,33,35,39,40,41,46,59,64,71,73,74,75,77,80]; (ii) only studies published in English were included; and (iii) the participant samples are not representative of the target population, being biased towards female, higher-educated, and younger adults whilst also lacking ethnical diversity.

## 5. Conclusions

The vital restrictions enforced in an endeavour to control the devastating effects of COVID-19 had a profound impact on PA and sedentary time/behaviour across the world [82]. The factors underpinning these effects are complex and multi-faceted. However, for adults, as we transition into a new normal, during any future periods of restrictions or as part of focused behaviour change interventions, targeting capability on a psychological level may be essential to both facilitate PA and limit sedentary time/behaviour. For PA, whilst factors such as social support and motivation may also be important, limiting restrictions to opportunity on a physical level could be crucial.

## Figures and Tables

**Table 1:** Study inclusion/exclusion criteria.

Variable	Inclusion Criteria	Exclusion Criteria
Population or participants and condition or interest	Adults aged 18 years or older Any sex/gender Not restricted to the UK	Studies including children and adolescents (aged less than 18 years)
Intervention or exposures	Exposure to the COVID-19 pandemic, containment, and mitigation strategies	Studies that involve non-COVID-19 related pandemics, such as SARS or MERS
Comparison or control groups	No restrictions	
Outcomes of interest	Data/information, qualitative or quantitative, relating to correlates of PA and/or sedentary time/behaviour during the COVID-19 pandemic	No data relating to the pandemic phase or restrictions in place available Studies only including empirical data on volume of or changes in volume of PA or sedentary time/behaviour Data pooled from multiple different countries
Setting	Any community setting	
Study designs	Any randomized, non-randomized, qualitative, or mixed methods study design providing original results	Studies not providing original results, such as systematic reviews, meta-analysis, general reviews, or editorials

COVID-19: novel coronavirus disease 2019; PA, physical activity; MERS, Middle East respiratory-system related coronavirus; SARS, severe acute respiratory syndrome; UK, United Kingdom.

**Table 2:** Illustrative summary of study characteristics and overall study quality.

		Number of Studies
Country of study	Australia	1 [62]
	Austria	1 [64]
	Bangladesh	2 [46,65]
	Belgium	1 [33]
	Brazil	3 [56,74,80]
	Canada	5 [32,48,49,61,67]
	Chile	1 [66]
	China	1 [51]
	Croatia	1 [42]
	France	1 [73]
	Ghana	1 [21]
	Hungary	1 [18]
	Japan	2 [60,78]
	Jordan	1 [19]
	KSA	1 [22]
	India	1 [44]
	Italy	5 [25,31,38,54,57]
	Northern Cyprus	1 [23]
	Spain	8 [20,26,27,28,29,52,53,72]
	Taiwan	1 [30]
	Thailand	1 [43]
	Turkey	1 [63]
	United Kingdom	9 [24,36,40,41,68,69,70,75,76]
	Ukraine	1 [71]
	USA	13 [34,35,37,39,45,47,50,55,58,59,77,79,81]
Study design	Observational	
	Cross-sectional	59 [18,19,20,21,22,23,24,25,26,27,28,30,31,32,33,34,35,36,37,38,40,41,42,43,45,46,47,48,49,50,51,52,53,54,56,57,58,59,60,61,62,63,64,65,66,67,68,69,70,71,73,74,75,76,77,78,79,80,81]
	Longitudinal	4 [29,39,55,72]
	Phenomenological	1 [44]
Correlated behaviour	Physical activity	64 [18,19,20,21,22,23,24,25,26,27,28,29,30,31,32,33,34,35,36,37,38,39,40,41,42,43,44,45,46,47,48,49,50,51,52,53,54,55,56,57,58,59,60,61,62,63,64,65,66,67,68,69,70,71,72,73,74,75,76,77,78,79,80,81]
	Sedentary behaviour	
	Active breaks	1 [66]
	Screen time	5 [19,32,58,59,80]
	Sitting time	11 [18,24,26,54,58,59,65,68,72,77,79]
	Sedentary time	4 [21,28,51,81]
Primary COVID-19 restrictions	Stay-at-home order	47 [18,19,20,21,22,23,24,25,26,27,28,29,31,34,35,36,38,40,41,42,43,44,45,46,51,52,53,54,55,56,57,60,62,63,64,65,68,69,70,71,72,73,74,75,76,78,80]
	Social distancing	4 [30,58,59,66]
	Varied by state/region	12 [32,37,39,47,48,49,50,61,67,77,79,81]
	Lockdown light	1 [33]
Overall study quality	*	1 [77]
	**	10 [31,55,56,57,59,60,71,73,74,75]
	***	29 [19,21,22,24,25,27,29,30,32,33,35,37,39,40,41,43,46,58,61,63,64,65,66,68,70,72,76,79,81]
	****	21 [18,20,23,26,28,34,38,42,45,47,48,49,50,51,52,53,54,67,69,78,80]
	*****	3 [36,44,62]

COVID-19: novel coronavirus disease 2019; KSA, Kingdom of Saudi Arabia; USA, United States of America. Overall study quality was assessed using the the Mixed Methods Assessment Tool (MMAT) and is reported using asterisks (*) as a descriptor, ranging from 1*, where 20% of the quality criteria have been met, to 5*, where 100% of the quality criteria have been met [15].

**Table 3:** Physical activity socioecological model themes mapped to the COM-B components.

Framework Theme	Theme	COM-B Component
Individual (biological)	General health	Capability (physical)
Individual (psychological)	Mental health	Capability (psychological)
Individual (psychological)	Motivation	Motivation (automatic)
Individual (psychological)	Cognitions	Capability (psychological)
Individual (psychological)	Behaviour	Motivation (reflexive)
Social	Sociodemographic factors	Opportunity (physical)
Social	Support	Opportunity (social)
Social	Domestic situation	Opportunity (social)
Social	Lifestyle choices	Capability (psychological)
Environment	Resources	Opportunity (physical)
Environment	Area of residence	Opportunity (physical)
Policy	COVID-19 related factors	Opportunity (physical)

COVID-19: novel coronavirus disease 2019.

## Data Availability

The data that support the findings of this study are available from the corresponding author upon reasonable request.

## Supplementary Materials

The following are available online at https://www.mdpi.com/article/10.3390/ijerph182010910/s1, Supplementary File S1: Further details of literature review stage methodology; Table S1: Study characteristics; Table S2: Coding criteria for the Mixed Methods Assessment Tool; Table S3: Quality assessment results from Mixed Methods Assessment Tool.
