# Supplementary material for: Moving Forward: Understanding Correlates of Physical Activity and Sedentary Behaviour during COVID-19—An Integrative Review and Socioecological Approach
Source: Int J Environ Res Public Health. 2021 Oct 17;18(20):10910. doi: 10.3390/ijerph182010910 (PMC8535281; doi:10.3390/ijerph182010910)
Supplement: Supplementary file 1 [file ijerph-18-10910-s001.zip › ijerph-1357598-GB-SI-supplementary RK KM.pdf]

Supplementary File S1. Further details of literature review stage methodology.

**Example of search strategy for SportDiscus**	TI "coronavirus" OR AB "coronavirus" OR TI "COVID-19" OR AB "COVID-19" OR TI "COVID 19" OR AB "COVID 19" OR TI "SARSCoV2" OR AB "SARSCoV2" OR TI "SARS-CoV-2" OR AB "SARS-CoV-2" OR TI "SARS CoV 2" OR AB "SARS CoV 2" OR TI "novel coronavirus" OR AB "novel coronavirus" OR TI "COVID" OR AB "COVID" OR TI "n-CoV" OR AB "n-CoV"<br>AND<br>TI "physical activ*" OR AB "physical activ*" OR TI "exercise" OR AB "exercise" OR TI "physical function" OR AB "physical function" OR TI "physical performance" OR AB "physical performance" OR TI "sport" OR AB "sport" OR TI "recreation" OR AB "recreation" OR TI "active travel" OR AB "active travel" OR TI "sedentary time" OR AB "sedentary time" OR TI "sedentary lifestyl*" OR AB "sedentary lifestyl*" OR TI "sedentary behavi*" OR AB "sedentary behavi*" OR TI "physical inactiv*" OR AB "physical inactiv*" OR TI " prolonged sitting" OR AB "prolonged sitting"<br>Terms were developed following Librarian guidance and extensive testing. The terms "sitting" and "screen time" were found to be encorporated within broader sedentary behavior/time term
**Study Selection Process**	Following the removal of duplicates, two authors (RLK and AWR) independently reviewed all generated citations and abstracts to select eligible studies using Rayyan (QRCI, Qatar), coding articles "Included" or "Excluded". Discrepancies were resolved through discussion. All articles subsequently coded "Included" were obtained as full-text articles and reviewed against the pre-defined inclusion/exclusion criteria independently by the two authors. Disagreements regarding eligibility were resolved by discussion with a third reviewer where necessary. Full text articles retrieved were hand searched via reference checking.
**Quality Assessment**	One reviewer (RLK) independently assessed study quality using the Mixed Methods Assessment Tool, a second reviewer (AWR) independently assessed 25% of the studies to ensure consistency. Disagreements between the two reviewers were resolved through discussion. No studies were excluded due to low quality, rather all issues were considered when interpreting the results of each study.
**Data extraction**	A standardised form based on the Cochrane Collaboration example was piloted and used to extract data from the included studies for evidence synthesis. Extracted information included: authors; year of publication; study design, setting, and population (including sample size, participant demographics and characteristics); study methodology, recruitment and completion rates (where applicable); inclusion/exclusion criteria; context of the pandemic (including stage, mitigation and confinement strategies in place at the time of the study), and, correlates of physical activity and sedentary behavior in relation to the COVID-19 pandemic. A second reviewer (AWR) independently reviewed 25% of the extracted data, discrepancies identified were resolved through discussion with a third reviewer (LS) where necessary. Supplementary data was consulted where available and necessary.

Table S1. Study characteristics.

Authors/ Year	Title	Country	Study Design	COVID-19 Restrictions	Inclusion/Exclusion Criteria	Aim	Participants	Pertinent Participant Demographics	Relevant Outcomes	Outcome Measurement Tools	Overall MMAT Grade
Acs et al. (2020) [18]	Effects of Covid-19 on Physical Activity Behaviour Among University Students: Results of A Hungarian Online Survey	Hungary	Cross-sectional, study via self-administered online survey	Home confinement	Student at University of Pecs	To examine and compare PA patterns among young adults before and during COVID-19 restrictions with respect to self-rated mental health and physical health during home confinement periods	n = 827<br>Mean age = 25.29 years (σ 8.09)	78.4% female<br>82.22% lived in urban environment<br>42.32% married or cohabiting<br>11.73% had children<br>37.6% did not participate in any sports pre-COVID-19	PA<br>Sitting time	IPAQ-SF	****
									Self-rated physical and mental health	5-point Likert scale (1 = worst to 5 = best)	
Alomari et al. (2020) [19]	Changes in Physical Activity and Sedentary Behavior Amid Confinement: The BKSQ-COVID-19 Project	Jordan	Cross-sectional, study via self-administered online survey	Home confinement/social distancing	Aged >18 years	To investigate changes in PA and SB during COVID-19	n = 1844<br>Mean age = 33.7 years (σ 11.3)<br>Range 18–72 years	69.5% female;<br>43.3% normal weight<br>34.5% low income<br>65.5% middle,<br>35.6% unemployed or retired<br>64.4 employed	PA<br>Screen time	Individually constructed questionnaire (Unvalidated)	***

Table S1. Study characteristics continued.

Authors/ Year	Title	Country	Study Design	COVID-19 Restrictions	Inclusion/Exclusion Criteria	Aim	Participants	Pertinent Participant Demographics	Relevant Outcomes	Outcome Measurement Tools	Overall MMAT Grade
Angosto et al. (2020) [20]	Motives and Commitment to Sport in Amateurs during Confinement: A Segmentation Study	Spain	Cross-sectional, Study via self-administered online survey	Stay-at-home order period into period of established time zones for outdoor PA based on age	Amateur sports persons over 18 years and resident in area of study	To assess the motives and commitment of the population to physical activity during the phase of confinement and return to the "new normality", identifying the latent factors in the motives and commitment. To compare the socio-demographic and motivational profiles according to the level of commitment to physical activity of the practitioner during the COVID-19 pandemic	n = 1025<br>Mean age = 35.3 years (σ 14.2)<br>Range 18–70 years	47.9% female;<br>72.7% at least graduate level educated<br>44% married or cohabiting<br>54.3% employed;<br>39.9% student;<br>5.7% unemployed or retired	Motives for PA	Individually constructed questionnaire (Validated)	****
Asiamah et al. (2021) [21]	Short-Term Changes in Behaviours Resulting from COVID-19-Related Social Isolation and Their Influences on Mental Health in Ghana	Ghana	Cross-sectional, study via self-administered online survey (convenience sample)	Mandatory stay-at-home order	General population of 3 cities, aged 18+ years, speak English, socially isolated due to lockdown.	To assess if changes in behaviours due to COVID-19 social distancing measures have a significant influence on mental health	n = 643<br>Mean age = 36 years<br>Range 18–64 years	35% female<br>94% had tertiary education	PA<br>Exercising<br>Sedentary time<br>Mental Health	Individually constructed questionnaire (Validated)<br>9-item Likert scale (1 = strongly disagree to 5 = strongly agree)	***

Table S1. Study characteristics continued.

Authors/ Year	Title	Country	Study Design	COVID-19 Restrictions	Inclusion/Exclusion Criteria	Aim	Participants	Pertinent Participant Demographics	Relevant Outcomes	Outcome Measurement Tools	Overall MMAT Grade
Barwais (2020) [22]	Physical activity at home during the covid-19 pandemic in the two most affected cities in Saudi Arabia	Kingdom of Saudi Arabia	Cross-sectional, study via self-administered online survey	24-hour curfew (stay-at home order)	Live in Mecca or Medina General population	To explore the amount of time people spend engaged in daily physical activity at home during the lockdown by examining differences in adults' levels of PA before and during the pandemic	*n* = 244 Mean age = 33.8 years (σ 7.7) Range 18–50 years	36.9% female Overall, 903 ± 755.6 MET-mins/week pre-COVID-19	PA	IPAQ-SF	***
Belgen et al. (2020) [23]	Determination of exercise habits, physical activity level and anxiety level of postmenopausal women during COVID-19 pandemic	Northern Cyprus	Cross-sectional, study via self-administered online survey (purposeful sample)	Self-quarantine/stay-at-home order	Postmenopausal women aged 50–75 years, with postmenopausal period of at least 1 year. Self-quarantined for 1 month	To investigate the exercise habits before and during the pandemic, PA levels and anxiety levels during the pandemic of post-menopausal women who were self-quarantined and did not go out of their houses for a month	*n* = 104 Mean age = 59 years (σ 6.62)	100% female 91.3% lived with at least 1 other person 52% doing regular exercise before the pandemic	PA<br>Anxiety	IPAQ-SF<br>Beck Anxiety Inventory	****

Table S1. Study characteristics continued.

Authors/ Year	Title	Country	Study Design	COVID-19 Restrictions	Inclusion/Exclusion Criteria	Aim	Participants	Pertinent Participant Demographics	Relevant Outcomes	Outcome Measurement Tools	Overall MMAT Grade
Brady et al. (2020) [24]	Different types of physical activity are positively associated with indicators of mental health and psychological wellbeing in rheumatoid arthritis during COVID-19	UK	Cross-sectional, study via self-administered online survey (purposeful sample)	Stay-at-home, once a day exercise permitted	Self-report clinical diagnosis of rheumatoid arthritis. Aged 18+ years	The aims of this study were to explore the associations between PA and ST with indicators of mental health and well-being in RA during COVID-19 and examine the impact of self-isolation on these associations	*n* = 345<br>Mean age = 51.48 years (σ 11.73)	93% female; 96.8% white; RA duration 10.52 ± 9.87 years	PA	National Institutes of Health- American Association of Retired Persons Diet and Health Study questionnaire	***
									Sitting time	IPAQ-SF	
									Pain	McGill Pain Questionnaire<br>Visual analogue scale- 0 (no pain) to 10 (worst imaginable)	
									Fatigue	Multidimensional Fatigue Inventory	
									Anxiety and Depression	HADS	
									Subjective Vitality	Subjective Vitality Scale	

Table S1. Study characteristics continued.

Authors/ Year	Title	Country	Study Design	COVID-19 Restrictions	Inclusion/Exclusion Criteria	Aim	Participants	Pertinent Participant Demographics	Relevant Outcomes	Outcome Measurement Tools	Overall MMAT Grade
Cancello et al. (2020) [25]	Determinants of the Lifestyle Changes during COVID-19 Pandemic in the Residents of Northern Italy	Italy	Cross-sectional, study via self-administered online survey	Stay-at-home order	18 years or over	To observe the effects of the lockdown on the reported lifestyle habits changes. The purpose of this survey was to give a picture of the Italian population perception of changes occurred in the main components of lifestyle (i.e., eating habits, PA, sleep, smoking) and to identify their determinants	*n* = 490	83.7% female; 58.4% high education level; 86.1% cohabiting; 14.5% 30 years or less; 65.1% 31–60; 20.4% >60 years; *n* = 272 active pre-lockdown	PA	Single question (Unvalidated)	***
Carriedo et al. (2020a) [26]	COVID-19, Psychological Well-being and Physical Activity Levels in Older Adults During the Nationwide Lockdown in Spain	Spain	Cross-sectional, study via self-administered online survey	Stay-at-home order	Aged 60 years or over. Living in Spain	To assess resilience, affect, depressive symptoms, and PA levels during the COVID-19 pandemic in Spanish older adults; and to evaluate the relationship between their psychological well-being and meeting PA recommendations	*n* = 483 Mean age = 65.49 years (σ 5.14) Range 60–92 years	No details	PA<br>Sitting time	IPAQ-SF	****
									Resilience	The Connor-Davidson CD-RISC resilience scale	
									Affect	Positive and Negative Affect Schedule	
									Depression	Adaptation of Six-item self-report scale (last 12-months)	

Table S1. Study characteristics continued.

Authors/ Year	Title	Country	Study Design	COVID-19 Restrictions	Inclusion/Exclusion Criteria	Aim	Participants	Pertinent Participant Demographics	Relevant Outcomes	Outcome Measurement Tools	Overall MMAT Grade
Carriedo et al. (2020b) [27]	Resilience and physical activity in people under home isolation due to COVID-19: A preliminary evaluation	Spain	Cross-sectional, study via self-administered online survey	Stay-at-home order	General population	To assess general Spanish population resilience and PA levels after the first week of the shelter-in-place health order issued, and to evaluate the relationship between resilience and PA levels	*n* = 1795<br>Mean age = 40.54 years (σ 15.68)<br>Range 16–82 years	64% female	PA	IPAQ-SF	***
									Resilience	The Connor-Davidson CD-RISC resilience scale	
Castaneda-Babarro et al. (2020) [28]	Physical Activity Change during COVID-19 Confinement	Spain	Cross-sectional, study via self-administered online survey	Stay-at-home order	Healthy adults Aged 18–64 years	To analyse self-reported PA and sedentary behaviour before and during lockdown caused by COVID-19 in a Spanish healthy adult population.	*n* = 3800<br>Mean age = 42.70 years (σ 10.4)<br>Range 18–64 years	46% female<br>7% students<br>78% employed; 0% study and work; 5% nothing	PA<br>Sedentary time	IPAQ-SF	****
Cecchini et al. (2021) [29]	A longitudinal study on depressive symptoms and physical activity during the Spanish lockdown	Spain	Longitudinal study via self-administered online survey	Stay-at-home order	General population	To measure prevalence and severity of depressive symptoms in Spanish confined people by collecting longitudinal data that assess their changes. To provide a concrete guide for PA in order to face this challenge efficiently and effectively	*n* = 595<br>Mean age = 45.60 years (σ 15.17)<br>Range 18–84 years	57.5% female<br>n = 482 (81previously physically active)	PA	IPAQ-SF	***
									Depression	Adaptation of Six-item self-report scale (last 12-months)	

Table S1. Study characteristics continued.

Authors/ Year	Title	Country	Study Design	COVID-19 Restrictions	Inclusion/Exclusion Criteria	Aim	Participants	Pertinent Participant Demographics	Relevant Outcomes	Outcome Measurement Tools	Overall MMAT Grade
Chang et al. (2020) [30]	Exercise Behavior and Mood during the COVID-19 Pandemic in Taiwan: Lessons for the Future	Taiwan	Cross-sectional, study via self-administered online survey	Social distancing/self-isolation, no formal lockdown	General population excluded if presence of COVID-19 symptoms or a positive diagnosis	To determine whether and how COVID-19 has affected exercise behaviour and mental health status in Taiwan. Additionally, a model was established to predict the changes in exercise behaviour during similar future pandemics, and potential moderators were examined	*n* = 1114<br>Mean age = 35.90 years (σ 15.16)	53.9% female<br>489 graduate level education or above.<br>1026 live urban, 86 rural	PA	2 Individually constructed questions (unvalidated)	***

Table S1. Study characteristics continued.

Authors/ Year	Title	Country	Study Design	COVID-19 Restrictions	Inclusion/Exclusion Criteria	Aim	Participants	Pertinent Participant Demographics	Relevant Outcomes	Outcome Measurement Tools	Overall MMAT Grade
Chirico et al. (2020) [31]	COVID-19 Outbreak and Physical Activity in the Italian Population: A Cross-Sectional Analysis of the Underlying Psychosocial Mechanisms	Italy	Cross-sectional, study via self-administered online survey	Stay-at-home order	General population	To survey the Italian population on its PA behaviour and how this latter was modelled by psychosocial variables during the emergency contingencies and measures taken for COVID-19 outbreak. Given the specific impact of the virus on the Lombardy region, a further aim was to evaluate specific differences between Lombardy inhabitants sample and the rest of the Italian population sample within the integrated model key variables	*n* = 2398 Mean age 31.84 years (σ 12.55)	57.6% females; 56.7% educated to degree or more; 85.9% cohabiting	Autonomous Motivation	Behavioural Regulation in Exercise Questionnaire version 3	**
									Theory of Planned Behaviour Constructs	Scale developed based on measures from previous studies covering: attitudes, subjective norms, perceived behavioural confidence and intention	
									Anxiety	State-Trait Anxiety Inventory	
									PA	Individually constructed question (unvalidated)	

Table S1. Study characteristics continued.

Authors/ Year	Title	Country	Study Design	COVID-19 Restrictions	Inclusion/Exclusion Criteria	Aim	Participants	Pertinent Participant Demographics	Relevant Outcomes	Outcome Measurement Tools	Overall MMAT Grade
Colley et al. (2020) [32]	Exercise and screen time during the COVID-19 pandemic	Canada	Cross-sectional study via self-administered online survey	Varied between states, from stay-at-home orders, to social distancing with variations on what facilities were closed.	Inclusion - general population aged 15 or over Excluded - living on reserves and Aboriginal settlements; full-time members of Canadian Armed Forces; institutionalised population; households in extremely remote areas with very low population density	To describe the exercise habits and changes to screen time behaviours among participants of the Canadian Perspectives Survey Series (CPSS) and to examine the relationships between these behaviours and self-perceived mental and general health.	*n* = 4524 Aged 20+	Not stated	Health and Mental Health<br>PA—Exercise habits<br>Screen time	Questions from Canadian Community Health Survey and Canadian Health Measures Survey	***
Constandt et al. (2020) [33]	Exercising in Times of Lockdown: An Analysis of the Impact of COVID-19 on Levels and Patterns of Exercise among Adults in Belgium	Belgium	Cross-sectional, study via self-administered online survey	Lockdown light. Schools closed, work from home where possible, encouraged to exercise at home and outdoors but alone, with family members or 1 friend. Forbidden to travel to exercise. All fitness centres closed	Resident of Flanders region, northern Belgium, aged 18–75 years	To examine the influence of the COVID-19 lockdown on adults' exercise behaviours in Flanders	*n* = 13,515 Range 18–75 years	50.5% female; 34.3% higher education degree; 94.4% high school degree or above; 39.2% had children at home	PA—Exercise	Individually constructed questionnaire (unclear if validated)	***

Table S1. Study characteristics continued.

Authors/ Year	Title	Country	Study Design	COVID-19 Restrictions	Inclusion/Exclusion Criteria	Aim	Participants	Pertinent Participant Demographics	Relevant Outcomes	Outcome Measurement Tools	Overall MMAT Grade
Coughenour et al. (2020) [34]	Changes in Depression and Physical Activity Among College Students on a Diverse Campus After a COVID-19 Stay-at-Home Order	USA (South Nevada)	Cross-sectional, study via self-administered online survey	State-wide stay-at-home order	Aged over 17 years, student at University of Nevada	To determine if there was a change in participants' PA mins prior to versus after stay-at-home orders were issued in the State of Nevada, to determine if there was a change in depression scores prior to versus after stay-at-home orders and understand predictors of depression score and PA changes among college students.	*n* = 194 Mean age = 25.11 years (σ 7.84)	72.2% female; High mean grade point average 0f 3.41 (SD 0.46); Ethnicity: White 18%; Asian 46%; Black 25%; Hispanic 34%; Other 17%	PA	Individually constructed questionnaire (Validated)	****
									Depression	Patient Health Questionnaire (PHQ-9)	

Table S1. Study characteristics continued.

Authors/ Year	Title	Country	Study Design	COVID-19 Restrictions	Inclusion/Exclusion Criteria	Aim	Participants	Pertinent Participant Demographics	Relevant Outcomes	Outcome Measurement Tools	Overall MMAT Grade
Dunca et al. (2020) [35]	Perceived change in physical activity levels and mental health during COVID-19: Findings among adult twin pairs	USA (Washington state)	Cross-sectional, study via self-administered online survey	Social distancing/stay-at-home	Identical and fraternal adult twins. Aged 18+ years	To investigate perceived changes in PA and mental health in response to COVID-19 mitigation strategies in identical and fraternal adult twins. To examine whether associations between perceived changes in PA and mental health were confounded by genetic and shared environmental factors	*n* = 3971 Mean age = 50.4 years (σ 16.0) *n* = 909 [77%] same-sex pairs)	69.2% female 95.5% white non-Hispanic 60.1% identical, 39.9% fraternal	PA/exercise	Single question (Unvalidated)	***
									Stress	Perceived Stress Scale	
									Anxiety	Brief Symptom Inventory	

Table S1. Study characteristics continued.

Authors/ Year	Title	Country	Study Design	COVID-19 Restrictions	Inclusion/Exclusion Criteria	Aim	Participants	Pertinent Participant Demographics	Relevant Outcomes	Outcome Measurement Tools	Overall MMAT Grade
Fallon et al. (2020) [36]	Adverse effects of COVID-19-related lockdown on pain, physical activity and psychological well-being in people with chronic pain	UK	Cross-sectional analytic study (Non-randomised) via self-administered online survey	Stay-at-home order, once a day exercise permitted	UK adult residents aged >18 years. Self-reported sufferer of chronic pain or non-pain for control group	To capture the effects of the COVID-19 pandemic, and corresponding UK lockdown restrictions, on pain, psychological wellbeing and PA levels in a group of participants suffering from chronic pain compared to a non-pain group	*n* = 519<br>Mean age = 43.98 years (σ 13.38)<br>Range 18–79 years	90.56% female<br>*n* = 431 chronic pain<br>*n* = 88 healthy controls	Pain	Visual analogue scales. Pain Catastrophizing Scale, an adapted version of the Brief pain Inventory	*****
									PA	Visual analogue scale	
Fearnbach et al. (2021) [37]	Factors Protecting against a Decline in Physical Activity during the COVID-19 Pandemic	USA	Cross-sectional, study via self-administered online survey	Varied between states—included stay-at-home orders, social distancing, closure of facilities	Adults aged 18 years or over with access to the internet	The purpose of this secondary analysis of the Pennington Biomedical COVID Health Behaviours Survey was to identify individual-level factors that helped people maintain PA levels amid the initial COVID-19 shutdown	*n* = 4376<br>Mean age = 52 years (σ 15)	Mean data<br>80% femal<br>81% household size >1.<br>Mean PA/week 348 mins (σ 403) or 1,453 (σ 1982) MET mins/week pre-COVID-19	PA	Long-form PA questionnaire modified from the Nurses Health PA Questionnaire (Validated)	***

Table S1. Study characteristics continued.

Authors/ Year	Title	Country	Study Design	COVID-19 Restrictions	Inclusion/Exclusion Criteria	Aim	Participants	Pertinent Participant Demographics	Relevant Outcomes	Outcome Measurement Tools	Overall MMAT Grade
Gallè et al. (2020) [38]	Sedentary Behaviours and Physical Activity of Italian Undergraduate Students during Lockdown at the Time of CoViD-19 Pandemic	Italy	Cross-sectional, study via self-administered online survey	Stay-at-home order	Student at 1 of 3 Italian universities	To evaluate the sedentary activities and PA levels the students assumed during home-confinement with respect to their previous habits, and to investigate the possible determinants of enduring PA during the lockdown	*n* = 1430 Mean age = 22.9 years (σ 3.5)	65.5% female 94.1% Italian 60.7% reside on campus. Mean PA = 520 min/week (SD 820) Mean SB = 240 min/day (SD 240) pre-COVID-19	PA	IPAQ-SF plus 3 additional questions	****
Gildner et al. (2021) [39]	Exercise routine change is associated with prenatal depression scores during the COVID-19 pandemic among pregnant women across the United States	USA (All states including Puerto Rico)	Longitudinal, study via self-administered online survey	Varied between states - included stay-at-home orders, social distancing, closure of facilities	Pregnant women, aged 18 years or over living in the USA	To assess how the COVID-19 pandemic has affected pregnant women's wellbeing	*n* = 1856 Mean age = 31.3 years (σ 4.30) Range 18–47 years	Weeks pregnant at time of survey 26.1 (σ 8.62, range 4–41) 87% white 55.2% household income > $100,000 77.1% degree educated or higher	Depression	Edinburgh Postnatal Depression Survey	***
									Exercise	Individually constructed questionnaire (Unvalidated)	

Table S1. Study characteristics continued.

Authors/ Year	Title	Country	Study Design	COVID-19 Restrictions	Inclusion/Exclusion Criteria	Aim	Participants	Pertinent Participant Demographics	Relevant Outcomes	Outcome Measurement Tools	Overall MMAT Grade
Ingram et al. (2020) [40]	Changes in Diet, Sleep, and Physical Activity Are Associated with Differences in Negative Mood During COVID-19 Lockdown	UK (Scotland)	Cross-sectional, study via self-administered online survey	Stay-at-home order, once a day exercise permitted with easing of restrictions at time 2—2 weeks later, meeting outside with one other household allowed and T3—2 weeks later, meeting with 2 households allowed outdoors shielding group restrictions lifted	Scottish adult or long term resident	To examine the associations between socio-demographic factors and COVID-induced changes, and health behaviours (changes in alcohol consumption, diet, sleep, and PA)	*n* = 399<br>Mean age = 32.4 years (σ 11.4)<br>Range 18–72 years	56.4% female. 81.2% live urban. 88% cohabiting	PA	Individually created scale (Unvalidated)	***
									Mood	abbreviated Profile of Mood State (POMS) scale	
Jacob et al. (2020) [41]	The relationship between physical activity and mental health in a sample of the UK public: A cross-sectional study during the implementation of COVID-19 social distancing measures	UK	Cross-sectional, study via self-administered online survey	Stay-at-home order, once a day exercise permitted	Adults aged 18 years or over, residing UK and social-distancing	The aim of the present study was to investigate the cross-sectional association between PA levels with depressive symptoms, anxiety symptoms, and positive mental well-being in a sample of the UK public social distancing owing to COVID-19	*n* = 902	63.8% female. 55.2% married 58.6% employed; 67% earn > 25k per year<br>50.1% of people 35-64 years. 31.6% 18–34. 18.3% 65 or more	Mental Health	Beck Anxiety and Depression Inventory	***
									Mental Well-being	The Short Warwick-Edinburgh Mental Well-being Scale	
									PA	Individually constructed questionnaire (Unvalidated)	

Table S1. Study characteristics continued.

Authors/ Year	Title	Country	Study Design	COVID-19 Restrictions	Inclusion/Exclusion Criteria	Aim	Participants	Pertinent Participant Demographics	Relevant Outcomes	Outcome Measurement Tools	Overall MMAT Grade
Karuc et al. (2020) [42]	Moderators of Change in Physical Activity Levels during Restrictions Due to COVID-19 Pandemic in Young Urban Adults	Croatia	Cross-sectional, study via self-administered online survey	Stay-at-home order	Previously enrolled in longitudinal study between 2014–2017	To investigate the moderators of change in PA level after 30 days of restrictions due to the COVID-19 pandemic in young urban adults	*n* = 91 Range 20–21 years old	65% female Mean age = 21.6 (σ 0.4).35% male, mean age = 21.5 (σ 0.3) MVPA (min/day) median female 120, median male 135 pre-COVID-19	PA	School Health Action, Planning, and Evaluation System (SHAPES) questionnaire. PA change in patterns assessed separately with 1-item question	****
Katewongsa et al. (2020) Only data relating to 2020 subset [43]	The effects of the COVID-19 pandemic on the physical activity of the Thai population: Evidence from Thailand's Surveillance on Physical Activity 2020	Thailand	Cross-sectional, study via self-administered online survey	Nationwide overnight curfew/Social distancing /facility closures/restricting public gatherings/encouraged to stay-at-home	Adults with internet access. Clear gender specification on Facebook profile	To compare PA level prior to and during the pandemic in Thailand. To document changes in the types and amount of PA among differing groups of the Thai population based on sociodemographic characteristics. To assess whether people exposed to the national campaign, had better PA outcomes during the pandemic	*n* = 4482 in (2020 data only) Range 18–64 years	46.6and female; 68.8% aged. 18–39 years 54.7% active pre-COVID-19	PA	Global Physical Activity Questionnaire	***

Table S1. Study characteristics continued.

Authors/ Year	Title	Country	Study Design	COVID-19 Restrictions	Inclusion/Exclusion Criteria	Aim	Participants	Pertinent Participant Demographics	Relevant Outcomes	Outcome Measurement Tools	Overall MMAT Grade
Kaur et al. (2020) [44]	Physical Fitness and Exercise During the COVID-19 Pandemic: A Qualitative Enquiry	India	Phenomenology—via semi-structured telephone interviews	Stay-at-home order	Aged 18 years or over, Previously gym dependent for exercise (45+ mins), continuing home workout for last 6 months. Excluded - irregular gym users, those practicing other exercises, any physical or psychological condition or on medication	To understand people's unique experiences during the period of lockdown due to COVID-19 and explore the ways in which regular exercise engagements helped them deal with the psychological and physical consequences of home confinement	*n* = 22<br>Mean age = 26.5 years<br>Range 19–34 years	9% female<br>19 single<br>15 living alone<br>All middle or higher class	PA experiences during lockdown	Semi-Structured telephone interviews	*****

Table S1. Study characteristics continued.

Authors/ Year	Title	Country	Study Design	COVID-19 Restrictions	Inclusion/Exclusion Criteria	Aim	Participants	Pertinent Participant Demographics	Relevant Outcomes	Outcome Measurement Tools	Overall MMAT Grade
Kaushal et al. (2020) [45]	Social Cognition and Socioecological Predictors of Home-Based Physical Activity Intentions, Planning, and Habits during the COVID-19 Pandemic	USA	Cross-sectional, study via self-administered online survey	Stay-at-home order	Aged over 18 years, proficient in English, residing in USA states with lockdown measures in place and gyms and recreation centres were closed	To integrate variables of the physical environment derived from socioecological models alongside constructs from traditional social cognition theories to predict home-based PA. To test the effects of 2 socioecological constructs, namely, availability and use of home exercise equipment and constructs from the IBC model on intentions and habits with respect to home-based PA during the COVID-19 pandemic	*n* = 429<br>Mean age = 47.1 years (σ 6.26)	60% female<br>53% in good or excellent health<br>55.1% degree or higher<br>52.9% married/common law<br>Mean PA = 231 mins/week (σ 116) pre-COVID-19	Theory of Planned Behaviour Constructs	Scale developed based on measures used in previous studies (expectations of PA in next 2 weeks)	****
									Planning	Scale developed based on measures used in previous studies	
									PA Habit	Self-report behaviour index scale	
									Autono-mous Motivation	Individually constructed questions	
									Ex Equipment availability	Individually constructed questions	
									Past PA Behaviour	Individually constructed questions (Validated)	

Table S1. Study characteristics continued.

Authors/ Year	Title	Country	Study design	COVID-19 Restrictions	Inclusion/Exclusion Criteria	Aim	Participants	Pertinent Participant Demographics	Relevant Outcomes	Outcome Measurement Tools	Overall MMAT Grade
Khan et al. (2020) [46]	The impact of COVID-19 pandemic on mental health and wellbeing among home-quarantined Bangladeshi students: A cross-sectional pilot study	Bangladesh	Cross-sectional, study via self-administered online survey	Stay-at-home order	College or University student, home-quarantined, able to speak Bengali, residing in Bangladesh during the outbreak	To assess the level of psychological impact of college and university students who are bound to be home-quarantined as widespread lockdown started	*n* = 505	37.23% female 96.63% unmarried; 78.42% aged 20–24 years	PA/Exercise	Individually constructed questionnaire (Unvalidated)	***
									Anxiety, Depression and Stress	Depression, Anxiety and Stress Scale	
									Event specific distress	Impact of Event Scale	
Knell et al. (2020) [47]	Health Behavior Changes During COVID-19 Pandemic and Subsequent" Stay-at-Home" Orders	USA	Cross-sectional, study via self-administered online survey	Varied between states—included stay-at-home orders, social distancing, closure of facilities	Aged >18 years, currently residing in USA	To describe how positive health behaviours (PA, sleep) and negative health behaviours (alcohol, tobacco, drug use) changed during a 6-8 week period. To understand who were more likely to change their health behaviours, and to describe reported reasons why participants were changing	*n* = 1809	67.4% female; 83.3% college graduates 39.8% 35–49 years old 65.6% reside in Texas	PA	IPAQ-SF	****
									Sleep Quality	Pittsburgh Sleep Quality Index	
									Substance Use	Behavioural Risk Factor Surveillance System	
									Impact of COVID-19	Individually constructed questions	

Table S1. Study characteristics continued.

Authors/ Year	Title	Country	Study design	COVID-19 Restrictions	Inclusion/Exclusion Criteria	Aim	Participants	Pertinent Participant Demographics	Relevant Outcomes	Outcome Measurement Tools	Overall MMAT Grade
Lebel et al. (2020) [48]	Elevated depression and anxiety symptoms among pregnant individuals during the COVID-19 pandemic	Canada	Cross-sectional, study via self-administered online survey	Varied between states, from stay-at-home orders, to social distancing with variations on what facilities were closed	Living in Canada, able to read and write English, having a confirmed pregnancy < 35 weeks' gestation	To determine the prevalence of anxiety and depression symptoms in pregnant people during the COVID-19 pandemic and identify potential resilience factors associated with lower symptom	*n* = 1987<br>Mean age = 32.4 years (σ = 4.2)<br>Range 18–48 years	100% female<br>69% degree or higher<br>Mean income $70–80,000 USD	Anxiety	PROMIS Anxiety	****
									Social support	Social support effectiveness questionnaire	
									PA	Godin PA Questionnaire	
Lesser et al. (2020) [49]	The Impact of COVID-19 on Physical Activity Behavior and Well-Being of Canadians	Canada	Cross-sectional, study via self-administered online survey	Varied between states, from stay-at-home orders, to social distancing with variations on what facilities were closed.	Aged over 19 years, Canadian resident	To gain an understanding of the impact of the global pandemic and public health restrictions on Canadians. Specifically, to report changes since COVID-19 began on PA barriers and facilitators and engagement, as well as well-being, in active and inactive individuals. To explore differences in outdoor PA and nature exposure based on classifications of generalised anxiety and well-being.	*n* = 1098<br>Mean age = 42 years (σ 15)	79.3% female<br>68.5% married/domestic partnership<br>33.5% degree or higher<br>76.8% employed<br>45% urban environment	PA	Godin PA Questionnaire and Individually constructed questions	****
									Motivation to exercise	Behavioural Regulations in Exercise Questionnaire	
									Outdoor PA	Nature relatedness scale	
									Anxiety	GAD-7	
									Well-being	Mental Health Continuum (MHC-SF)	

Table S1. Study characteristics continued.

Authors/ Year	Title	Country	Study design	COVID-19 Restrictions	Inclusion/Exclusion Criteria	Aim	Participants	Pertinent Participant Demographics	Relevant Outcomes	Outcome Measurement Tools	Overall MMAT Grade
Limbers et al. (2020) [50]	Physical activity moderates the association between parenting stress and quality of life in working mothers during the COVID-19 pandemic	USA	Cross-sectional, study via self-administered online survey	Varied between states - included stay-at-home orders, social distancing, closure of facilities	English speaking woman from USA, aged 18 years or older, at least 1 child 5 years old or younger living with them at least 50% of time; working minimum of 30hours/week- normally outside home but now working at home full time.	To 1) evaluate the associations between parenting stress, quality of life, and PA in a national sample of working mothers transitioned to working from home due to the pandemic, and 2) examine if PA moderates the association between parenting stress and quality of life in this sample	*n* = 200<br>Mean age = 33.5 years (σ 6.25)<br>Range 19–60 years	100% female<br>75% married<br>78% day care or childcare closed<br>80.5% degree or higher	PA	IPAQ - SF	****
									QoL	WHOQOL - BREF	
									Parenting Stress	Parental Stress Scale	

Table S1. Study characteristics continued.

Authors/ Year	Title	Country	Study design	COVID-19 Restrictions	Inclusion/Exclusion Criteria	Aim	Participants	Pertinent Participant Demographics	Relevant Outcomes	Outcome Measurement Tools	Overall MMAT Grade
Lin et al. (2020) [51]	Depression is Associated with Moderate-Intensity Physical Activity Among College Students During the COVID-19 Pandemic: Differs by Activity Level, Gender and Gender Role	China	Cross-sectional, study via self-administered online survey	Lockdown policies implemented in almost all regions	Chinese college student	To investigate associations between depression and PA among Chinese college students and to examine the effect of gender and gender role on such relationships during the COVID-19 pandemic	*n* = 625 Mean age = 20.18 years (no σ)	64.8% female	PA	IPAQ-SF	****
									Sedentary time	IPAQ -SF	
									Depression	Center for Epidemiological Studies Depression Scales (CES-D)	
									Gender Role	Chinese Sex-Role Inventory	
Lopez-Bueno, Calatayud, Andersen et al. (2020) [52]	Immediate Impact of the COVID-19 Confinement on Physical Activity Levels in Spanish Adults	Spain	Cross-sectional, study via self-administered online survey	Stay-at-home order	Spanish adults	To quantify the change in PA of the Spanish adult population during COVID-19 confinement compared with before confinement. Quantification of this change is important to provide practical recommendations, e.g., on physical activities to perform at home	*n* = 2042 Mean age 35.9% years (σ 13.6)	54% female 49% married 63% university degree Mean PA = 221.9 mins/week (σ 193.6) pre-COVID-19	PA	Physical activity vital sign (PAVS) short form	****

Table S1. Study characteristics continued.

Authors/ Year	Title	Country	Study design	COVID-19 Restrictions	Inclusion/Exclusion Criteria	Aim	Participants	Pertinent Participant Demographics	Relevant Outcomes	Outcome Measurement Tools	Overall MMAT Grade
Lopez-Bueno, Calatayud, Ezzatvar et al. (2020) [53]	Association Between Current Physical Activity and Current Perceived Anxiety and Mood in the Initial Phase of COVID-19 Confinement	Spain	Cross-sectional, study via self-administered online survey	Stay-at-home order	Spanish adults	The main objective of this study was to evaluate the association between current PA and current perceived both anxiety and mood among Spanish adults confined due to COVID-19	*n* = 2250 Mean age 35.3 years (SD 13.6)	54.8% female 38.9% meeting WHO guidelines prior to COVID-19	PA	Physical activity vital sign (PAVS) - SF	****
									Perceived anxiety	Individually constructed questions	
									Perceived mood	Individually constructed questions	
Luciano et al. (2020) [54]	COVID-19 lockdown: Physical activity, sedentary behaviour and sleep in Italian medicine students	Italy	Cross-Sectional longitudinal study via self-administered online survey	Stay-at-home order	1st to 6th year medical students	To describe PA, sedentary behaviour and sleep of Italian medicine students during COVID-19 lockdown	n = 1470 Mean age = 23 years (σ 2)	70% female	PA	IPAQ-SF	****
									Sitting time	IPAQ-SF	
									Sleep Quality	Pittsburgh Sleep Quality Index	
Maher et al. (2021) [55]	Physical activity is positively associated with college students' positive affect regardless of stressful life events during the COVID-19 pandemic	USA (North Carolina)	Longitudinal study via self-administered online survey	Stay-at-home order	College students	To determine associations between PA and affect among college students before and during COVID-19 stay-at-home orders and how change in PA predicts change in affect during this time. To determine whether stressful life events during stay-at-home orders moderated association between PA and affect	*n* = 107 Mean age = 21.7 years (σ 2.6) Range 18–34 years	71.3% female 54% white	Affect	Positive and Negative Affect Schedule	**
									PA	IPAQ - SF	
									Sleep Quality	Pittsburgh Sleep Quality Index	
									Stressful life events	Social Re-adjustment Rating Scale	
									Mental Well-being	HADS	

Table S1. Study characteristics continued.

Authors/ Year	Title	Country	Study Design	COVID-19 Restrictions	Inclusion/Exclusion Criteria	Aim	Participants	Pertinent Participant Demographics	Relevant Outcomes	Outcome Measurement Tools	Overall MMAT Grade
Martinez et al. (2020) [56]	Physical activity in periods of social distancing due to COVID-19: a cross-sectional survey	Brazil	Cross-sectional, study via self-administered online survey	Social distancing and stay-at-home order	18 years or older: ability to understand Portuguese, born/currently live in Brazil; practised physical exercise until pre-COVID-19 restrictions	To assess the changes in the habits of the Brazilian population engaged in PA in relation to their practices, due the measures of social distancing. As a secondary objective, we sought to describe the levels of anxiety and depression of this population, during the period of social distancing	*n* = 1613 Mean age females = 35.4 years (σ 12) Mean age males = 36.2 years (σ 10.9)	63.1% female; 75.3% Higher education 86.9% cohabiting; 44.5% married; 81% good self-perception of health 45.7% active 5 or more days per week pre-COVID-19	PA	Individually constructed questionnaire (Unvalidated)	**
Maugeri et al. (2020) [57]	The impact of physical activity on psychological health during Covid-19 pandemic in Italy	Italy	Cross-sectional, study via self-administered online survey	Stay-at-home order	No details	To examine (1) changes in the PA levels during self-quarantine in Italy; (2) the correlation between PA and well-being of individuals	*n* = 2524	56.4% female. Split into 4 age groups Aged <21 (*n* = 346); young adult aged 21–40 (*n* = 1178); adults aged 41–60 (*n* = 1704) and over 60 age (*n* = 296)	PA	IPAQ - SF	**
									Psychological and general well-being	Psychological and General Well Being Index	
									Motivation to exercise	Behavioural Regulations in Exercise Questionnaire	
									Anxiety	GAD-7	
									Well-being	Mental Health Continuum (MHC-SF)	

Table S1. Study characteristics continued.

Authors/ Year	Title	Country	Study Design	COVID-19 Restrictions	Inclusion/Exclusion Criteria	Aim	Participants	Pertinent Participant Demographics	Relevant Outcomes	Outcome Measurement Tools	Overall MMAT Grade
McDowell et al. (2020) [58]	Working from Home and Job Loss Due to the COVID-19 Pandemic Are Associated With Greater Time in Sedentary Behaviours	USA (Iowa)	Cross Sectional survey - via self-administered online survey (purposeful sample)	Social distancing/self-quarantine/closure of facilities	Adult, employed prior to COVID-19	To explore whether COVID-19-related employment changes were associated with activity behaviours, including sitting time, screen time, and PA, in a sample of 2,303 previously employed USA adult participants in the COVID-19 and Wellbeing Study	n = 2303	66% female Data split between groups for different work patterns no overall summary. Primarily Caucasian	PA	IPAQ	***
									Sitting time	Unclear—inferred probable question from IPAQ	

Table S1. Study characteristics continued.

Authors/ Year	Title	Country	Study Design	COVID-19 Restrictions	Inclusion/Exclusion Criteria	Aim	Participants	Pertinent Participant Demographics	Relevant Outcomes	Outcome Measurement Tools	Overall MMAT Grade
Meyer et al. (2020) [59]	Changes in Physical Activity and Sedentary Behavior in Response to COVID-19 and Their Associations with Mental Health in 3052 US Adults	USA (Iowa)	Cross-sectional, study via self-administered online survey	Social distancing/self-quarantine/closure of facilities	Aged ≥18 years; current USA resident	To evaluate the impact of COVID-19-related public health guidelines on PA, sedentary behaviour, mental health, and their interrelations	n = 3052	62% female; 93.1% Caucasian; 67.6% married or relationship	Mental Health	Perceived Stress Scale-4<br>Loneliness scale<br>The Short Warwick-Edinburgh Mental Well-being Scale<br>Lubben Social Network Scale-6<br>Beck Depression Inventory<br>Beck Anxiety Inventory	**
									PA	Individually constructed question (unclear if validated)	
									Sitting time and screen time	Individually constructed question (unclear if validated)	

Table S1. Study characteristics continued.

Authors/ Year	Title	Country	Study Design	COVID-19 Restrictions	Inclusion/Exclusion Criteria	Aim	Participants	Pertinent Participant Demographics	Relevant Outcomes	Outcome Measurement Tools	Overall MMAT Grade
Munekawa et al. (2020) [60]	Effect of coronavirus disease 2019 pandemic on the lifestyle and glycaemic control in patients with type 2 diabetes: a cross-section and retrospective cohort study	Japan	Cross-sectional, study via self-administered online survey	Stay-at-home order	Included - Type 2 Diabetes Mellitus. Excluded - hospital admission or changed diabetes medication during past 3 months	To investigate the acute effects of the COVID-19 pandemic on the lifestyle changes in patients with type 2 diabetes mellitus. Furthermore, we also investigated the association between these changes and metabolic parameters, including body weight and haemoglobin A1c levels.	*n* = 183 Mean age = 67.4 years (σ 11.3)	62.1% male	Stress and lifestyle factors (including exercise/PA)	Visual analogue scale (0 = considerably reduced, 5 = no change, and 10 = considerably increased)	**
Nienhuis et al. (2020) - linked to Lesser [61]	The Impact of COVID-19 on Women's Physical Activity Behavior and Mental Well-Being	Canada	Cross-sectional, study via self-administered online survey	Varied between states, from stay-at-home orders, to social distancing with variations on what facilities were closed	Aged 19 years or over	To assess whether sex differences exist in PA and well-being since COVID-19 and to explore how barriers or facilitators to PA may explain these differences. To look at whether changes in occupation or childcare responsibilities since COVID-19 have impacted women's well-being and PA engagement	*n* = 1086	80.1% female, mean age = 41 (σ 15); 215 males, mean age = 45 (σ 16). Greater % of women higher educated greater % of men in full time work	PA	Godin PA Questionnaire + additional questions	***
									Motivation to exercise	Behavioural Regulations in Exercise Questionnaire	
									Anxiety	GAD-7	
									Well-being	Mental Health Continuum (MHC-SF)	

Table S1. Study characteristics continued.

Authors/ Year	Title	Country	Study Design	COVID-19 Restrictions	Inclusion/Exclusion Criteria	Aim	Participants	Pertinent Participant Demographics	Relevant Outcomes	Outcome Measurement Tools	Overall MMAT Grade
Oliva et al. (2020) (look at for qual data display) [62]	Puppy love in the time of Corona: Dog ownership protects against loneliness for those living alone during the COVID-19 lockdown	Australia	Mixed methods cross-sectional study via self-administered online survey	Stay-at-home order	Aged 18 years or older, Australian citizens, living alone	To capture the experience of the COVID-19 lockdown in an Australian population living alone. Specifically, to investigate whether pet owners living alone demonstrate higher levels of mindfulness as compared to non-owners living alone while adjusting for previous mindfulness training experiences	*n* = 384 Mean age = 50.92 years (σ 15.09 years)	85.4% female; 69% degree education or higher; 66% self-isolating	Impact of having a pet on COVID 19 experience	Two open ended questions	*****
Ozdemir et al. (2020) [63]	The role of physical activity on mental health and quality of life during COVID-19 outbreak: A cross-sectional study	Turkey	Cross-sectional, study via self-administered online survey	Stay-at-home order	Included - No details. Excluded -psychiatric or neurological diseases	To evaluate the PA levels of individuals and assess the effects of PA on quality of life, depression and anxiety levels during COVID-19 outbreak.	*n* = 2301 Mean age =36.2 (σ 10.9) Range 20–75 years	61.1% female; 61% aged 20-39; 59.6% married; 72.5% employed	PA	IPAQ - SF	***
									Depression	Beck Depression Inventory	
									Anxiety	Beck Anxiety Inventory	
									QoL	WHOQOL - BREF	
									Active breaks		

Table S1. Study characteristics continued.

Authors/ Year	Title	Country	Study Design	COVID-19 Restrictions	Inclusion/Exclusion Criteria	Aim	Participants	Pertinent Participant Demographics	Relevant Outcomes	Outcome Measurement Tools	Overall MMAT Grade
Pieh et al. (2020) [64]	The effect of age, gender, income, work, and physical activity on mental health during coronavirus disease (COVID-19) lockdown in Austria	Austria	Cross-sectional, study via self-administered online survey	Stay-at-home order, exercise outdoors alone permitted	General population	To evaluate mental health in a representative adult sample in Austria after 4 weeks of lockdown considering relevant influencing factors such as age, gender, income, and job situation.	n = 1005	52.7% female; 36.7% university educated	PA	Individual question (Unvalidated)	***
									Mental Health	WHOQOL-BREF<br>WHO-5 well-being<br>Perceived Stress (PSS-10)<br>Depression (PHQ-9)<br>GAD-7	
Rahman et al. (2020) [65]	Physical inactivity and sedentary behaviours in the Bangladeshi population during the COVID-19 pandemic: An online cross-sectional survey	Bangladesh	Cross-sectional, study via self-administered online survey	Stay-at-home order	Bangladeshi residence, Aged 18 years or older, able to read Bangla.	To determine the prevalence of physical inactivity and sedentary behaviours during the COVID-19 pandemic among Bangladeshi people.	n = 2038<br>Mean age = 25.9 years (σ 8.1)<br>Range 18–65 years	42.8% female<br>71% single<br>64.4% degree or above	PA	IPAQ - SF	***
									Sitting time	IPAQ - SF	

Table S1. Study characteristics continued.

Authors/ Year	Title	Country	Study Design	COVID-19 Restrictions	Inclusion/Exclusion Criteria	Aim	Participants	Pertinent Participant Demographics	Relevant Outcomes	Outcome Measurement Tools	Overall MMAT Grade
									PA		

Table S1. Study characteristics continued.

Authors/ Year	Title	Country	Study Design	COVID-19 Restrictions	Inclusion/Exclusion Criteria	Aim	Participants	Pertinent Participant Demographics	Relevant Outcomes	Outcome Measurement Tools	Overall MMAT Grade
Rhodes et al. (2020) [67]	Correlates of Perceived Physical Activity Transitions during the COVID-19 Pandemic among Canadian Adults	Canada	Cross-sectional, study via self-administered online survey	Varied between states, from stay-at-home orders, to social distancing with variations on what facilities were closed.	English speaking; aged 18 years or over	To (1) replicate the change in MVPA findings from China, Europe, and the United States, within a Canadian sample, (2) explore correlates of current MVPA and shifts in MVPA after the COVID-19 restrictions had been in place for nearly two months, and (3) predict the stability of the MVPA profiles (e.g., inactive throughout, active throughout, those who were active but became inactive) as a result of the COVID-19 pandemic restrictions.	n = 1055 Mean age = 48.82 (σ 16.66)	51% female 83% white; 29% responsible for children; 48.9% degree or higher 48.2% full time employment; 28.1% dog owner Mean PA = 201.38 (σ 223.76) mins per week pre-COVID-19	PA	Godin PA Questionnaire	****
									Psychological factors / personality trait factors	NEO 5 factor Inventory; Neuroticism scale; Executive Function Index sub-scale.	
									Social factors	Study created items	
									Home environment	Based on home exercise environment affordances measure (Sallis et al.1997)	
									Neighbourhood environment	Neighbourhood walkability scale<br>International PA Prevalence Study Environmental Survey Module	

Table S1. Study characteristics continued.

Authors/ Year	Title	Country	Study Design	COVID-19 Restrictions	Inclusion/Exclusion Criteria	Aim	Participants	Pertinent Participant Demographics	Relevant Outcomes	Outcome Measurement Tools	Overall MMAT Grade
Richardson et al. (2020) [68]	The influence of COVID-19 measures in the United Kingdom on physical activity levels, perceived physical function and mood in older adults: A survey-based observational study	UK	Cross-sectional, convergent mixed-methods, observational Survey via self-administered online survey	Stay-at-home, once a day exercise permitted	At least 70 years old, living in the UK, absent of any cognitive disorders, with access to the internet	To examine the impact that COVID-19 measures in the UK, had on individuals aged 70 and over in terms of their PA levels, perceived physical functioning, and mood	*n* = 117<br>Mean age = 75 years (σ 4)	55.6% females<br>All Caucasian<br>*n* = 82 married or cohabiting<br>Higher than average activity levels	PA<br>Sitting time	IPAQ-Elderly	***
									Perception of function and disability	Late-life Function and Disability Instrument	
									Mood	Brunel Mood Scale (BRUMS)	
Robinson et al. (2021) [69]	Obesity, eating behavior and physical activity during COVID-19 lockdown: A study of UK adults	UK	Cross-sectional, study via self-administered online survey	Stay-at-home, once a day exercise permitted	Aged 18 years or over, fluent English, UK resident	To examine perceived changes (before vs. during social lockdown) to a range of weight-related behaviours in a large sample of UK adults during social lockdown, as well as to examine whether there have been common barriers to weight management because of the COVID-19 crisis	*n* = 2002<br>Mean age = 34.74 years (σ 12.3)	61.7% female; 65.5% degree level education; 89.7% white	Perceived behaviour changes	Individually constructed questions	****
									PA	IPAQ	
									Diet quality	Food frequency questionnaire	
									Overeating	Addiction-Like Eating Behavior Scale	
									Well-being	WHO-5 well-being	

Table S1. Study characteristics continued.

Authors/ Year	Title	Country	Study Design	COVID-19 Restrictions	Inclusion/Exclusion Criteria	Aim	Participants	Pertinent Participant Demographics	Relevant Outcomes	Outcome Measurement Tools	Overall MMAT Grade
Rogers et al. (2020) [70]	Behavioral Change Towards Reduced Intensity Physical Activity Is Disproportionately Prevalent Among Adults With Serious Health Issues or Self-Perception of High Risk During the UK COVID-19 Lockdown	UK	Cross-sectional convergent mixed method study via self-administered online survey	Stay-at-home, once a day exercise permitted	Aged 13 years or over, living in UK	To identify whether the UK's lockdown measures have had disproportionate impacts on PA intensity in groups who are, or who perceive themselves to be at risk of worse outcomes of COVID-19 disease	*n* = 9190<br>Range 20–70+ years	77.7% female<br>82% aged 35–69<br>62% educated to degree level or higher<br>95% white	PA	Individually constructed questionnaire (Validated)	***
									Disability	Individually constructed questionnaire	
									Depression	Individually constructed questionnaire	
Rogowska et al. (2020) [71]	Does Physical Activity Matter for the Mental Health of University Students during the COVID-19 Pandemic?	Ukraine	Cross-sectional, study via self-administered online survey	Stay-at-home-order and social distancing	Ukranian student at 1 of 18 universities	To examine the prevalence of anxiety and depression disorders during the pandemic in a sample of university students in Ukraine.	*n* = 1512<br>Mean age = 20.06 years (σ 3.05)<br>Range 18-51 years	68.65% female;<br>32.67% rural<br>46.49% meeting PA guidelines pre-COVID-19	PA	Individually constructed questionnaire (Unvalidated)	**
									Anxiety	GAD-7	

Table S1. Study characteristics continued.

Authors/ Year	Title	Country	Study Design	COVID-19 Restrictions	Inclusion/Exclusion Criteria	Aim	Participants	Pertinent Participant Demographics	Relevant Outcomes	Outcome Measurement Tools	Overall MMAT Grade
Romero-Blanco et al. (2020) [72]	Physical Activity and Sedentary Lifestyle in University Students: Changes during Confinement Due to the COVID-19 Pandemic	Spain	Longitudinal study via self-administered online survey	Stay-at-home order	1st-4th year Health Science University Student	To evaluate students' PA and sedentary behaviour at two points in time: before and during the coronavirus lockdown. Also, to look at changes in PA and sedentary behaviour resulting from other factors such as alcohol and tobacco consumption, adherence to a Mediterranean diet, motivation, symptoms of anxiety/depression and sociodemographic characteristics	*n* = 213 Mean age = 20.5 years (σ 4.6)	80.8% female; 76.1% cohabiting: Pre-COVID-19 PA - Pre-contemplation 1.9%, Contemplation 11.3%, Preparation 23.9%, Action 41.3%, Maintenance 21.6%	Perceived health status including anxiety and depression	EQ-5D	***
									PA	IPAQ - SF	
									Stages of change in PA	Transtheoretical Model	
									Sitting time	IPAQ - SF	
Rossinot et al. (2020) [73]	Behavioral Changes During COVID-19 Confinement in France: A Web-Based Study	France	Cross-sectional, study via self-administered online survey	Stay-at-home order	French speaking, living in France, initially no age restrictions then limited to 24-65 years old	To measure, characterise and understand the determinants of the negative impact of confinement on health-related behaviours and the mental state of the French adult population	*n* = 1454 Range 24–65 years	63.5% female 69.3% increased education 80% access to outdoor space	PA	Part of individually constructed questionnaire (Unvalidated)	**
									Mental Health	Individual question	
									Other outcomes	Individualised questionnaire	

Table S1. Study characteristics continued.

Authors/ Year	Title	Country	Study Design	COVID-19 Restrictions	Inclusion/Exclusion Criteria	Aim	Participants	Pertinent Participant Demographics	Relevant Outcomes	Outcome Measurement Tools	Overall MMAT Grade
Silva et al. (2020) [74]	Physical Inactivity Is Associated with Increased Levels of Anxiety, Depression, and Stress in Brazilians During the COVID-19 Pandemic: A Cross-Sectional Study	Brazil	Cross-sectional, study via self-administered online survey	Social distancing and stay-at-home order	No details	To evaluate the levels of anxiety, depression, and stress associated with the practice of exercise during COVID-19 outbreak	*n* = 1154<br>Mean age = 31.15 years (σ 9.68)<br>30.32% of the participants not practicing any type of exercise before social isolation	69.84% female<br>34.83% married<br>At least 40.2% degree level of higher	Physical Exercise practice before and during pandemic	Individual question (Unvalidated)	**
									Depression	Depression Anxiety Stress Scales 21 (DASS- 21	
Smith et al. (2020) [75]	Prevalence and correlates of physical activity in a sample of UK adults observing social distancing during the COVID-19 pandemic	UK	Cross-sectional, study via self-administered online survey	Stay-at-home, once a day exercise permitted	Aged 18 years and over, residing in the UK, observing social distancing	To examine the levels of physical activity during the UK COVID-19 social distancing guidance and investigate how such levels vary by sociodemographic, behavioural, clinical and contextual factors	*n* = 911	64% female<br>50.4% aged 35–64 years only<br>18.3% over 65	PA	Individually constructed questionnaire (unvalidated)	**

Table S1. Study characteristics continued.

Authors/ Year	Title	Country	Study Design	COVID-19 Restrictions	Inclusion/Exclusion Criteria	Aim	Participants	Pertinent Participant Demographics	Relevant Outcomes	Outcome Measurement Tools	Overall MMAT Grade
Spence et al. (2020) [76]	Determinants of physical activity among adults in the United Kingdom during the COVID-19 pandemic: The DUK-COVID study	UK	Cross-sectional, study via self-administered online survey	Stay-at-home, once a day exercise permitted	Aged 18 years or older, UK resident	To survey current PA, changes in PA modalities (i.e., overall, around the home, for transport, in the workplace, in the local neighbourhood, at recreation/sport facilities) related to the lockdown, and potential motivational determinants of such behaviour among adults residing in the UK.	*n* =1521	86% white<br>51% female<br>68.7% 30–64 years old	PA	Individually constructed questionnaire (Validated)	***
Stephan et al. (2020) [77]	Physical Activity and Sedentary Behavior During COVID-19: Trajectory and Moderation by Personality	USA	Cross-sectional, study via self-administered online survey	Varied between states - included stay at home orders, social distancing, closure of facilities	No details	To examine the change in both PA and sedentary behaviour during the COVID-19 pandemic and whether personality is associated with these changes	*n* = 6136<br>Mean age = 48.62 years<br>(σ 16.08)	58% female<br>78% white	COM-B beliefs	6-item scale adapted from Keyworth, Epton, Goldthorpe, Calam, and Armitage (2020)	*
									PA	Individual question (Unvalidated)	
					Aged 18 years or older, living in USA		*n* = 2230<br>Mean age = 43.75<br>(σ 18.64)	54% female<br>62% white	Personality	Big Five Inventory-2	
									Sitting time	Individual question (Unvalidated)	

Table S1. Study characteristics continued.

Authors/ Year	Title	Country	Study Design	COVID-19 Restrictions	Inclusion/Exclusion Criteria	Aim	Participants	Pertinent Participant Demographics	Relevant Outcomes	Outcome Measurement Tools	Overall MMAT Grade
Suzuki et al. (2020) [78]	Physical Activity Changes and Its Risk Factors among Community-Dwelling Japanese Older Adults during the COVID-19 Epidemic: Associations with Subjective Well-Being and Health-Related Quality of Life	Japan	Cross-sectional study via 2 types of mailed out self-administered questionnaires	Stay-at-home order	Aged 65 years old or over	To understand the impact of public health restrictions on community-dwelling older adults since the onset of COVID-19, in relation to the changes in PA, subjective well-being and HRQoL. To investigate the personal, social, and physical environmental factors linked to a decline in PA	*n* = 165 Mean age = 78.6 years (σ 8)	69.7% female (Large demographic profile presented in the paper)	PA	Physical Activity Questionnaire for Elderly Japanese (PAQ-EJ) plus additional question	****
									Functional Health	Tokyo Metropolitan Institute of Gerontology Index of Competence	
									Neighbourhood environment	International Physical Activity Questionnaire Environment	
									Subjective Well-Being	WHO-5 well-being	
									HRQoL	Medical Outcome Study	

Table S1. Study characteristics continued.

Authors/ Year	Title	Country	Study Design	COVID-19 Restrictions	Inclusion/Exclusion Criteria	Aim	Participants	Pertinent Participant Demographics	Relevant Outcomes	Outcome Measurement Tools	Overall MMAT Grade
Weaver et al. (2021) [79]	Health Behaviours at the Onset of the COVID-19 Pandemic	USA	Cross-sectional, study via self-administered online survey	Varied between states - included stay at home orders, social distancing, closure of facilities	Aged 18 years or over, English speaking, living in the USA	To assess perceived changes to health behaviours before and during COVID-19 restrictions and identify modifiable and non-modifiable risk/protective factors associated with health behaviours.	*n* = 362 Range 18–60+ years	51.1% married; 68.8% white; 81.8% no children 62.8% degree educated or higher Mean PA = 2205 (σ 3342.7) met mins/week pre-COVID-19	PA	IPAQ-SF	***
									Sitting time		
									Diet quality	Dietary Screening Questionnaire	
									Social Isolation	Individual question	
									Self-rated health	Valid single item	
									Resilience	Valid scale	
									Depression	Center for Epidemiological Studies Depression Scales	
									Perceived social support	Multidimensional Scale of Perceived Social Support	
									Subjective Well-Being	Valid single item	

Table S1. Study characteristics continued.

Authors/ Year	Title	Country	Study Design	COVID-19 Restrictions	Inclusion/Exclusion Criteria	Aim	Participants	Pertinent Participant Demographics	Relevant Outcomes	Outcome Measurement Tools	Overall MMAT Grade
Werneck et al. (2020) [80]	The mediation role of sleep quality in the association between the incidence of unhealthy movement behaviours during the COVID-19 quarantine and mental health	Brazil	Cross-sectional, study via self-administered online survey	Social distancing and stay at home order	General population, aged 18 years or over	To investigate the mediating role of worsening sleep quality in association with incidence of physical inactivity, high TV-viewing and high computer/tablet use with loneliness, sadness and anxiety	*n* = 45,161 (no summary stats)	*n* = 16,059 for PA incidence, 40,903 for tv viewing and 20,752 for computer/tablet use	PA<br>Screen time<br>Sleep Quality<br>Mental Health	Individually constructed questionnaire (Unvalidated)	****
Yang et al. (2020) [81]	Determinants of physical activity maintenance during the Covid-19 pandemic: a focus on fitness apps	USA (45 states)	Cross-sectional study via self-administered online survey	Varied between states - included stay at home orders, social distancing, closure of facilities	Healthy adults, aged 18–65 years, English speaking, own a smartphone, have downloaded at least one PA app	To consider gamification-related features besides motivational and educational features of PA apps as factors to describe three relevant clusters of app features that might help predict the maintenance of PA.	*n* = 431<br>Mean age = 39.1 years (σ 10.6)	49% female;<br>68.7% degree educated or higher<br>82.1 % Caucasian<br>84% employed<br>75% aged 21–45 years<br>Mean PA = 3323 met mins/week (σ 2451) pre-COVID-19	PA<br>Sedentary time<br>PA app use	IPAQ - SF (PA, Sedentary time)<br>Individual questions (PA app use)	***

COVID-19 = novel coronavirus disease 2019; EQ-5D = EuroQol-5 dimension; GAD-7 = general anxiety disorder-7; HADS = hospital anxiety and depression scale; HRQoL = health-related quality of life; IPAQ = International Physical Activity Questionnaire; QoL = quality of life; MET = metabolic equivalent; mins = minutes; MMAT = mixed methods assessment tool; MVPA = moderate-to-vigourous physical activity; n = number; PA = physical activity; PROMIS = patient-reported outcomes measurement information system; RA = rheumatoid arthritis; SB = sedentary behaviour; SD = standard deviation; SES = socioeconomic status; SF = short form; UK = United Kingdom; USA = United States of America; WHO = World Health Organization.

Table S2. Coding criteria for the Mixed Methods Assessment Tool.

**Code Breakdown**	
Screening questions (for all types)	S1. Are there clear research questions?
	S2. Do the collected data allow to address the research questions?
1. Qualitative	1.1. Is the qualitative approach appropriate to answer the research question?
	1.2. Are the qualitative data collection methods adequate to address the research question?
	1.3. Are the findings adequately derived from the data?
	1.4. Is the interpretation of results sufficiently substantiated by data?
	1.5. Is there coherence between qualitative data sources, collection, analysis and interpretation?
2. Quantitative randomized controlled trials	2.1. Is randomization appropriately performed?
	2.2. Are the groups comparable at baseline?
	2.3. Are there complete outcome data?
	2.4. Are outcome assessors blinded to the intervention provided?
	2.5 Did the participants adhere to the assigned intervention?
3. Quantitative non-randomized	3.1. Are the participants representative of the target population?
	3.2. Are measurements appropriate regarding both the outcome and intervention (or exposure)?
	3.3. Are there complete outcome data?
	3.4. Are the confounders accounted for in the design and analysis?
	3.5. During the study period, is the intervention administered (or exposure occurred) as intended?
4. Quantitative descriptive	4.1. Is the sampling strategy relevant to address the research question?
	4.2. Is the sample representative of the target population?
	4.3. Are the measurements appropriate?
	4.4. Is the risk of nonresponse bias low?
	4.5. Is the statistical analysis appropriate to answer the research question?
5. Mixed methods	5.1. Is there an adequate rationale for using a mixed methods design to address the research question?
	5.2. Are the different components of the study effectively integrated to answer the research question?
	5.3. Are the outputs of the integration of qualitative and quantitative components adequately interpreted?
	5.4. Are divergences and inconsistencies between quantitative and qualitative results adequately addressed?
	5.5. Do the different components of the study adhere to the quality criteria of each tradition of the methods involved?

Table S3. Quality assessement results from Mixed Methods Assessment Tool.

Authors / Year (Location)	1. Qualitative Studies					2. Randomized Controlled Trials					3. Non-Randomized Studies					4. Quantitative Descriptive Studies					5. Mixed Methods Studies					Overall Score	Comments
	1.1.	1.2.	1.3.	1.4.	1.5.	2.1.	2.2.	2.3.	2.4.	2.5.	3.1.	3.2.	3.3.	3.4.	3.5.	4.1.	4.2.	4.3.	4.4.	4.5.	5.1.	5.2.	5.3.	5.4.	5.5.		
Acs et al. (2020) [18]																Y	Y	Y	U	Y						4	No other identifiable issues
Alomari et al. (2020) [19]																Y	Y	N	U	Y						3	4.3 unvalidated outcome measurement.
Angosto et al. (2020) [20]																Y	Y	Y	U	Y						4	Non-prob sampling but specifically targeting amateurs
Asiamah et al. (2020) [21]																Y	N	Y	U	Y						3	4.2 Sampling method bias highly educated sample not representative of targeted population, low mean age
Barwais (2020) [22]																Y	N	Y	U	Y						3	4.2 Target population not clearly defined, if it is general population then mean age is low
Belgen et al. (2020) [23]																Y	Y	Y	U	Y						4	? Should be paired t-tests not independent as only 1 group
Brady et al. (2020) [24]																Y	N	Y	U	Y						3	4.2 93% of sample female
Cancello et al. (2020) [25]																Y	Y	N	U	Y						3	4.3 No validation of PA measurement
Carriedo et al. (2020a) [26]																Y	Y	Y	U	Y						4	No other identifiable issues
Carriedo et al. (2020b) [27]																Y	U	Y	U	Y						3	4.2 Minimal participant details and no inclusion/exclusion criteria provided
Castaneda-Babarro et al. (2020) [28]																Y	Y	Y	U	Y						4	No other identifiable issues
Cecchini et al. (2021) [29]																Y	N	Y	U	Y						3	4.2 Based on their comments on page 3, stating that the sample may not be representative
Chang et al. (2020) [30]																Y	Y	N	U	Y						3	4.3 No validation of PA assessment tool. Categorical data
Chirico et al. (2020) [31]																Y	N	N	U	Y						2	4.2 Low mean sample age, not representative of the population. 4.3 Unvalidated measure of PA

Colley et al. (2020) [32]																Y	U	Y	U	Y	3	4.2 No participant demographics
Constandt et al. (2020) [33]																Y	Y	U	U	Y	3	4.3 ?No validation of outcome measurements
Coughenour et al. (2020) [34]																Y	Y	Y	N	Y	4	4.4 Risk of Nonresponse bias as predominantly high achieving respondents
Dunca et al. (2020) [35]																Y	Y	N	U	Y	3	4.3 Unvalidated measure of PA
Fallon et al. (2020) [36]											Y	Y	Y	Y	Y						5	3.3 yes for data we are looking at 3.4 see section 3.3 of paper
Fearnbach et al. (2021) [37]																Y	N	Y	U	Y	3	4.2 80% female
Gallè et al. (2020) [38]																Y	Y	Y	U	Y	4	No other identifiable issues
Gildner et al. (2021) [39]																Y	Y	N	U	Y	3	4.3 Unvalidated measure of PA
Ingram et al. (2020) [40]																Y	N	N	U	Y	3	4.2 mean age low but ? No is too harsh 4.3 unvalidated method of PA measurement but appropriate for what is measuring?
Jacob et al. (2020) [41]																Y	Y	N	U	Y	3	4.3 unvalidated method of PA measurement but appropriate for what is measuring?
Karuc et al. (2020) [42]																Y	Y	Y	N	Y	4	4.4 Response bias analysis pg 3 Stats appropriate for question but ? Why median not mean data
Katewongsa et al. (2020) [43]																Y	N	Y	U	Y	3	4.2 Sample is bias towards younger participants
Kaur et al. (2020) [44]	Y	Y	Y	Y	Y																5	No identifiable issues
Kaushal et al. (2020) [45]																Y	Y	Y	U	Y	4	No other identifiable issues
Khan et al. (2020) [46]																Y	Y	N	U	Y	3	4.3 unvalidated measure of PA but not their primary outcome
Knell et al. (2020) [47]																Y	Y	Y	U	Y	4	No other identifiable issues

Lebel et al. (2020) [48]						Y	Y	Y	U	Y	4	No other identifiable issues
Lesser et al. (2020) [49]						Y	Y	Y	U	Y	4	No other identifiable issues
Limbers et al. (2020) [50]						Y	N	Y	U	Y	4	4.2 highly educated, predominantly white, married participants
Lin et al. (2020) [51]						Y	Y	Y	U	Y	4	No other identifiable issues
Lopez-Bueno et al. (2020a) [52]						Y	Y	Y	U	Y	4	No other identifiable issues
Lopez-Bueno et al. (2020b) [53]						Y	Y	Y	U	Y	4	No other identifiable issues
Luciano et al. (2020) [54]						Y	Y	Y	U	Y	4	Sample is difficult to assess as there are 2 different samples
Maher et al. (2021) [55]						N	N	Y	U	Y	2	4.1 & 4.2 Sampling strategy is limited to one specific class and therefore not representative
Martinez et al. (2020) [56]						Y	N	N	U	Y	2	4.3 unvalidated measure of PA 4.2 low population mean, sample not representative
Maugeri et al. (2020) [57]						U	U	Y	U	Y	2	4.1 & 4.2 No inclusion/exclusion criteria stated therefore unclear who the target population is
McDowell et al. (2020) [58]						Y	N	Y	U	Y	3	4.2 sample predominantly Caucasian, educated, married
Meyer et al. (2020) [59]						Y	N	U	U	Y	2	4.2 sample predominantly Caucasian, educated, married 4.3 Minimum info on measurements
Munekawa et al. (2020) [60]						Y	N	N	N	Y	2	4.2 Small selective sample 4.3 unvalidated measure of PA 4.4 limited to those who chose to attend clinic during a pandemic, high chance those who did not attend more stressed
Nienhuis et al. (2020) [61]						Y	N	Y	U	Y	3	4.2 aim to look at differences between sexes but sample predominantly female
Oliva et al. (2020) [62]	Y	Y	Y	Y	Y						5	Study mixed method but only qualitative component relevant for this review therefore mixed methods criteria not assessed. Although support for the 1

																											specific correlate of interest is low 23/108 dog owner's overall quality of study has no identifiable concerns
Ozdemir et al. (2020) [63]																Y	N	Y	U	Y						3	4.2 sample bias towards younger participants plus no detail on inclusion criteria
Pieh et al. (2020) [64]																Y	Y	U	U	Y						3	no clear detail of method of determining PA
Rahman et al. (2020) [65]																Y	N	Y	U	Y						3	4.2 - low mean sample age
Reyes-Olavarría et al. (2020) [66]																Y	N	Y	U	?Y						3	Survey pilot tested 4.2; low median age
Rhodes et al. (2020) [67]																Y	Y	Y	U	Y						4	No identifiable issues
Richardson et al. (2020) [68]																Y	N	Y	U	Y						3	Even though mixed methods, Only assessed on quantitative descriptive as qualitative data not applicable to the review outcomes I.e. not related to correlates of PA 4.2 homogenous sample with similar SES and ethnicity
Robinson et al. (2021) [69]																Y	Y	Y	U	Y						4	No other identifiable issues
Rogers et al. (2020) [70]	Y	Y	Y	Y	Y											Y	N	Y	U	Y	N	Y	Y	Y	N	3	4.3 questionnaire validated prior to use 5.1 no rationale provided 4.2 sample is bias towards educated, white, females 5.1 no justification for mixed method design. Overall score limited by quantitative descriptive parameters
Rogowska et al. (2020) [71]																Y	N	N	U	Y						2	4.2 could presume sample is representative but no inclusion criteria detailed 4.3 Unvalidated measure of PA
Romero-Blanco et al. (2020) [72]																Y	N	Y	U	Y						3	4.2 bias towards female students and only sampled from one course
Rossinot et al. (2020) [73]																Y	N	N	U	Y						2	4.2 no inclusion/exclusion criteria but then under 24 year olds and over 65 year olds removed 4.3 unvalidated measure of PA
Silva et al. (2020) [74]																Y	N	N	U	Y						2	4.2 no inclusion criteria plus low sample mean age. 4.3 unvalidated measure of PA

Smith et al. (2020) [75]	Y	N	N	U	Y	2	4.2 sample bias towards younger participants 4.3 unvalidated measure of PA
Spence et al. (2020) [76]	Y	N	Y	U	Y	3	4.2 sample ethically bias
Stephan et al. (2020) [77]	U	N	N	U	Y	1	4.1 unclear who the target population is limited/no inclusion criteria. 4.2 sample biased to white, educated… 4.3 no validation of outcome measure of PA or SB. 4.4 unclear.
Suzuki et al. (2020) [78]	Y	Y	Y	N	Y	4	4.4 of 400 targeted only 165 included
Weaver et al. (2021) [79]	Y	N	Y	U	Y	3	4.2 whilst sample stratified it is not ethnically diverse
Werneck et al. (2020) [80]	Y	Y	N	Y	Y	4	4.3 unvalidated questionnaire
Yang et al. (2020) [81]	Y	N	Y	N	Y	3	4.2 87% Caucasian 4.4 49% attrition rate

Y = yes; N = no; U = unclear.
